# Prescription of therapeutic exercise in migraine, an evidence-based clinical practice guideline

**DOI:** 10.1186/s10194-023-01571-8

**Published:** 2023-06-07

**Authors:** Roy La Touche, José Fierro-Marrero, Irene Sánchez-Ruíz, Borja Rodríguez de Rivera-Romero, Carlos Donato Cabrera-López, Sergio Lerma-Lara, Néstor Requejo-Salinas, Francisco de Asís-Fernández, Ignacio Elizagaray-García, Josué Fernández-Carnero, Luís Matesanz-García, Joaquín Pardo-Montero, Alba Paris-Alemany, Álvaro Reina-Varona

**Affiliations:** 1 Departamento de Fisioterapia, Centro Superior de Estudios Universitarios La Salle, Universidad Autónoma de Madrid, Aravaca, Madrid, Spain; 2 Motion in Brains Research Group, Centro Superior de Estudios Universitarios La Salle, Universidad Autónoma de Madrid, Aravaca, Madrid, Spain; 3 PhD Program in Medicine and Surgery. Doctoral School, Universidad Autónoma de Madrid, Madrid, Spain; 4 Instituto de Dolor Craneofacial y Neuromusculoesquelético (INDCRAN), Madrid, Spain; 5 Research Group Breatherapy, Centro Superior de Estudios Universitarios La Salle, Universidad Autónoma de Madrid, Aravaca, Madrid, Spain; 6 CranioSPain Research Group, Centro Superior de Estudios Universitarios La Salle, 28023 Madrid, Spain; 7 Department of Physical Therapy, Occupational Therapy, Rehabilitation and Physical Medicine, Rey Juan Carlos University, 28922 Alcorcón, Spain; 8 Department of Radiology, Rehabilitation and Physiotherapy. Faculty of Nursery, Physiotherapy and Podiatry, Complutense University of Madrid, Madrid, Spain

**Keywords:** Migraine disorders, Exercise therapy, Clinical practice guideline, Migraine symptoms, Disability, Quality of life

## Abstract

The main objective of this clinical practice guideline is to provide a series of recommendations for healthcare and exercise professionals, such as neurologists, physical therapists, and exercise physiologists, regarding exercise prescription for patients with migraine.

This guideline was developed following the methodology and procedures recommended in the Appraisal of Guidelines for Research and Evaluation (AGREE). The quality of evidence and strength of recommendations were evaluated with the Scottish Intercollegiate Guidelines Network (SIGN). A systematic literature review was performed and an established appraisal process was employed to rate the quality of relevant scientific research (Grading of Recommendations Assessment, Development, and Evaluation methodology).

The evaluation of the current evidence, the elaboration of the grades of recommendation, and their validation show a B grade of recommendation for aerobic exercise, moderate-continuous aerobic exercise, yoga, and exercise and lifestyle recommendations for the improvement of symptoms, disability, and quality of life in patients with migraine. Relaxation techniques, high-intensity interval training, low-intensity continuous aerobic exercise, exercise and relaxation techniques, Tai Chi, and resistance exercise obtained a C grade of recommendation for the improvement of migraine symptoms and disability.

**Supplementary Information:**

The online version contains supplementary material available at 10.1186/s10194-023-01571-8.

## Introduction

Migraine is the second leading cause of disability in the world after low back pain [1]. It is a neurological condition with a global prevalence of 14.4%, with the peak prevalence and years of life lived with disability occurring between ages 35 and 39 years [2]. The impact generated on the patient’s various social and family dimensions induces a decline in quality of life and a high economic cost due to work absenteeism, a decrease in work efficiency, and increased healthcare costs [1, 3, 4].

Pharmacological interventions are the most common approaches, typically consisting of the use of non-steroidal anti-inflammatory drugs and triptans for acute management [5, 6]. Prophylactic drugs, such as topiramate or valproic acid, are recommended for chronic migraine [5, 6]. Other pharmacological approaches have been developed in recent years, including the human monoclonal antibody erenumab, botulinum toxin, ditans, and gepants, with good results in the reduction of the frequency of migraine and evolution of migraine attacks [7–9]. However, the main problem with these interventions is their concomitant adverse effects, such as the increase in blood pressure with triptans use, the risk of allergic reaction with monoclonal antibodies, the transient development of blepharoptosis and muscle weakness produced by the injection of botulinum toxin, and interaction with other drugs [6–10].

In addition to pharmacological treatment, behavioral change interventions are fundamental in the clinical management of migraine. These treatments include management of stress, sleep, diet, and exercise [11, 12], of which aerobic exercise and yoga modalities are proposed as preventive alternatives for migraine [12]. Exercise prescription for migraine improvement appears to be a safe and effective intervention that could decrease migraine symptoms and disability and increase quality of life. Aerobic exercise has been the most studied modality [13, 14].

Migraine and exercise information disseminated on social networks has increased exponentially in recent years; however, the quality of that information is questionable [15]. Several international scientific societies recommend the practice of exercise as part of the therapeutic approach to migraine. The French Headache Society includes physical exercise as part of the non-pharmacological treatment of migraine headaches [16]; the Danish Headache Society agrees with a similar recommendation and also includes relaxation and postural exercises [17]; and the American Headache Society recommends regular exercise as part of the biobehavioral treatment of migraine management and prevention [18].

The recommendations of the various headache societies for treatments involving exercise for migraine have one characteristic in common: exercise is mentioned in a very general way, and the various exercise modalities that can be used for migraine treatment are not mentioned in depth. Current scientific evidence has not yet determined the adequate exercise prescription parameters for patients with migraine. Also, there are still no clinical practice guidelines on exercise prescription for migraine. Therefore, we consider it necessary to develop a guide to help clinicians who treat headaches so they can make better recommendations or provide a more specific exercise prescription.

The main objective of this clinical practice guideline is to provide a series of recommendations regarding different exercise modalities that could be effective in the treatment of migraine, and other lifestyle recommendations that could increase the efficacy of exercise interventions, for healthcare and exercise professionals, such as neurologists, physical therapists, and exercise physiologists, so as to better treat patients with migraine. For this proposal, we reviewed the current evidence that shows which exercise interventions improve migraine symptoms (intensity, frequency, and duration), disability, and quality of life. Moreover, the intention of this guideline is to provide parameters of exercise prescription for each exercise modality that could be adapted depending on the patient’s characteristics (e.g. migraine frequency, physical condition, and patient’s preferences). It is not a standard of medical care that determines the exercise intervention approach for migraine treatment. Patients’ clinical presentation, experiences, and expectations, as well as clinicians’ experiences and expertise should guide the exercise prescription based on the best recommendations of the current evidence.

## Methods

### Overall design and organization

Content experts were appointed by the Institute of Neuroscience and Sciences of Movement (INCIMOV) from the La Salle University Center for Advanced Studies (CSEULS) to conduct a systematic review for the development of clinical practice guidelines regarding exercise prescription for patients with migraine. The guideline was reported in accordance with the Reporting Items for Practice Guidelines in Healthcare (RIGHT) statement [19], and the Appraisal of Guidelines, Research and Evaluation (AGREE) checklist [20] was consulted to ensure the quality of the guideline.

### Funding and support

The Professional College of Physiotherapists of the Community of Madrid provided funding and support for this clinical practice guideline. This institution did not take part in the development of the recommendations.

### Guideline working group

The task force for the Evidence-Based Practice Guidelines for Exercise Prescription in Migraine Patients consisted of 3 groups: an advisory committee and panel, an expert consensus group, and a scientific evidence evaluation group. Task force members came from a wide range of disciplines, including medicine, physiotherapy, physical activity and sport sciences, and psychology. The scientific-technical knowledge and skills of the task force were related to exercise prescription, migraine diagnosis and treatment, evidence-based medicine, and research methodology.

### Registration and protocol

The present clinical practice guideline was registered in the Practice guideline REgistration for transPAREncy (PREPARE) with the registration number PREPARE-2023CN046.

### Literature search

A systematic review of the evidence regarding exercise efficacy for improving the symptoms, disability, and quality of life in patients with migraine was performed. This review was elaborated in accordance with the Preferred Reporting Items for Systematic Reviews and Meta-analyses (PRISMA) checklist [21]. Moreover, this study was previously registered in PROSPERO, an international register for systematic reviews (CRD42022316319).

### Search strategy

The search strategy combined medical subject headings (MeSH) and non-MeSH terms and was applied to the following databases without language or time restrictions: MEDLINE (PubMed), Cochrane, EBSCO, Web of Science, and Google Scholar. The most important terms were “Migraine” and “Exercise,” and the last search was conducted in December 2022. Various sub-searches were developed due to the variety of exercise modalities available for the treatment of migraine. The search strategy information is available in the Supporting Information Appendix S1.

Two independent reviewers conducted the search using the same methodology. If any difference emerged during this phase, it was resolved by consensus. Moreover, original articles were manually screened, and the authors were contacted for further information if necessary.

### Selection criteria and data extraction

Systematic reviews, randomized controlled trials, quasi-experimental trials, cohort and case–control designs, case series, case reports, and narrative reviews were screened and included in this review. Any form of study that evaluated the effects of exercise on the symptoms, disability, and quality of life of patients with migraine was of relevance for the development of the present clinical practice guidelines.

A specified list of inclusion and exclusion criteria was elaborated for the screening of articles based on the Population, Intervention, Comparator and Outcome (PICO) measure model [22]. The inclusion criteria for the participants in the articles included were patients with episodic or chronic migraine, diagnosed by a physician based on any of the International Classification of Headache Disorders (ICHD) editions [23], and age 18 years or older. The intervention must be or include exercise in any modality (e.g., aerobic, yoga, resistance training), and the comparator could be any other form of evidence-based exercise intervention that has been shown to be effective for migraine, placebo, or waiting list. Finally, the outcome measures included were pain intensity; migraine attack frequency, defined mainly as days with migraine per month; and duration of migraine attacks, evaluated primarily as the number of hours per migraine attack. Disability and quality of life measures were also analyzed.

For the selection criteria and data extraction, 2 independent reviewers examined the title, abstract, and keywords of each article using the inclusion and exclusion criteria. A full-text article review was similarly conducted for the final elaboration of the set of articles included for the clinical practice guideline recommendations. If any difference emerged during this phase, it was resolved by discussion, mediated by a third reviewer [24].

### Methodological quality and risk of bias assessment

Two independent reviewers assessed the methodological quality of the studies included in the review. Systematic reviews were evaluated with the Modified Quality Assessment Scales for Systematic Reviews (AMSTAR), developed by Barton et al. [25]. The Physiotherapy Evidence Database (PEDro) scale was used for the assessment of the randomized controlled trials and the quasi-experimental trials [26]. Cohort studies were evaluated with the Newcastle–Ottawa Quality Assessment Scale (NOS) [27]. For the evaluation of the case series studies, we employed the National Institutes of Health (NIH) Study Quality Assessment Tool for Case Series Studies [28]. Finally, we assessed the methodological quality of the narrative reviews with the Scale for the Assessment of Narrative Review Articles (SANRA) [29]. We also assessed the risk of bias in the systematic reviews and randomized controlled trials. The Risk of Bias in Systematic Reviews (ROBIS) tool was used for the evaluation of the systematic reviews, and the Cochrane revised Risk of Bias 2.0 scale (RoB 2.0) was used for the evaluation of the randomized controlled trials and the quasi-experimental studies [30, 31].

The inter-rater reliability between the 2 reviewers was evaluated with κ. This statistic shows a low level of agreement if κ < 0.5; κ of 0.5–0.7 shows a moderate level of agreement; and κ > 0.7 shows a high level of agreement [32]. If any disagreement appeared in the quality assessment score, it was resolved by consensus, mediated by a third independent reviewer.

### Level of evidence and grades of recommendation

Once the methodological quality and risk of bias assessments were performed, the Scottish Intercollegiate Guidelines Network (SIGN) was used to evaluate each study’s level of evidence and to determine the recommendation grade for each exercise intervention. This tool was designed for the development of evidence-based clinical guidelines, and it has a series of advantages: the methodological quality of each study determines the level of evidence; guideline developers must consider the generalizability, applicability, and consistency of each intervention; the clinical impact of the evidence creates a clear link between the evidence and the recommendation; and grades of recommendation are based on the strength of the supporting evidence, taking into account its overall level and the considered judgment of the guideline developers [33]. Table 1 shows the criteria for the levels of evidence and grades of recommendation.

**Table 1:** Score criteria for SIGN levels of evidence and grades of recommendation

Levels of evidence		Grades of recommendation	
**1 + + **	High-quality meta-analysis, systematic reviews of RCTs or RCTs with very low risk of bias	A	At least one meta-analysis, systematic review or RCT rated as 1 + + and directly applicable to the target population OR
**1 + **	Well-conducted meta-analyses, systematic reviews of RCTs or RCTs with low risk of bias		A systematic review of RCTs or a body of evidence consisting principally of studies rated as 1 + directly applicable to the target population and demonstrating overall consistency of results
**1-**	Meta-analyses, systematic reviews or RCTs, or RCTs with high risk of bias	B	A body of evidence including studies rated as 2 + + directly applicable to the target population and demonstrating overall consistency of results OR
**2 + + **	High-quality systematic reviews of case–control or cohort studies or High-quality case–control or cohort studies with a very low risk of confounding, bias or chance and a high probability that the relationship is causal		Extrapolated evidence from studies rated as 1 + + or 1 +
**2 + **	Well-conducted case–control or cohort studies with a low risk of confounding, bias or chance and a moderate probability that the relationship is causal	C	A body of evidence including studies rated as 2 + directly applicable to the target population and demonstrating overall consistency of results OR
**2-**	Case–control or cohort studies with a high risk of confounding, bias or chance and a significant risk that the relationship is not causal		Extrapolated evidence from studies rated as 2 + +
**3**	Non-analytic studies, e.g., case reports, case series	D	Evidence level 3 or 4 OR
**4**	Expert opinion		Extrapolated evidence from studies rated as 2 +

For the development of each intervention summary, we introduced “improve” or “decrease” (grade A), “likely to” (grade B), “might” (grade C), or “remotely” (grade D) depending on the grade of recommendation and the sum of studies that support or negate each intervention efficacy based on the various migraine variables (symptoms, disability, and quality of life). For example, if an intervention achieved a B grade of recommendation and 3 or more studies found a positive effect on pain intensity, it “is likely to decrease pain intensity”. However, if this same intervention had only 1 study that found a positive effect on quality of life, it “remotely improve quality of life”.

### Patient diagnosis

Subgroups of patients with migraine were established in the present clinical practice guideline to distinguish between episodic and chronic migraine. The ICHD defines chronic migraine as a headache occurring on 15 or more days per month for more than 3 months, which, on at least 8 days per month, has the features of migraine headache [23]. If the headache and migraine features are of lower frequency, it is considered an episodic migraine. This distinction is important, given that studies regarding exercise interventions on patients with migraine could include one or both diagnostics and influence the results obtained.

### Exercise modalities

We provided operational definitions for the various exercise modalities and multimodal interventions of this clinical practice guideline. These operational definitions summarize the main characteristics of the various interventions.

For the general exercise recommendations, we focused on the data from the systematic reviews published, and for the specific exercise modalities recommendations we focused on the available RCTs.

### Guideline review process and validation

For the evaluation and validation of the guidelines’ content, a panel of experts was organized. This panel, as mentioned in the guideline working group section, consisted of 8 physical therapists, 4 of whom were also physical activity and sports professionals, a physician, and a psychologist, all with extensive clinician and research experience in the treatment of patients with migraine and exercise prescription. Operational definitions of each intervention, methodological quality, risk of bias, level of evidence, recommendation grade, prescription parameters, and intervention summary were shown to the panel of experts in a presentation during a meeting in June 2022. The experts had to validate these various intervention categories. For this validation process, the experts used a 5-point Likert scale: (1) strongly disagree, (2) somewhat disagree, (3) neither agree nor disagree, (4) somewhat agreement, (5) strongly agree. Moreover, they could add any correction or suggestion to the various categories. After some rounds of deliberation, the experts reached a consensus and determined the validity of each modality and category.

### Updates

The procedure for updating the clinical practice guidelines will be structured according to the Checklist for the Reporting of Updated Guidelines [34] and by analyzing the amount and relevance of emerging evidence for exercise prescription in patients with migraine.

## Results

### Study selection

**Fig. 1:**
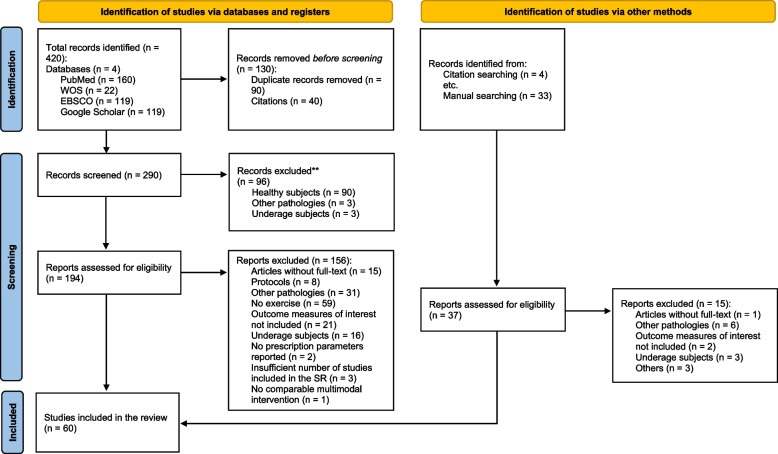
Flow chart of the study selection and inclusion process according to PRISMA

**Fig. 2:**
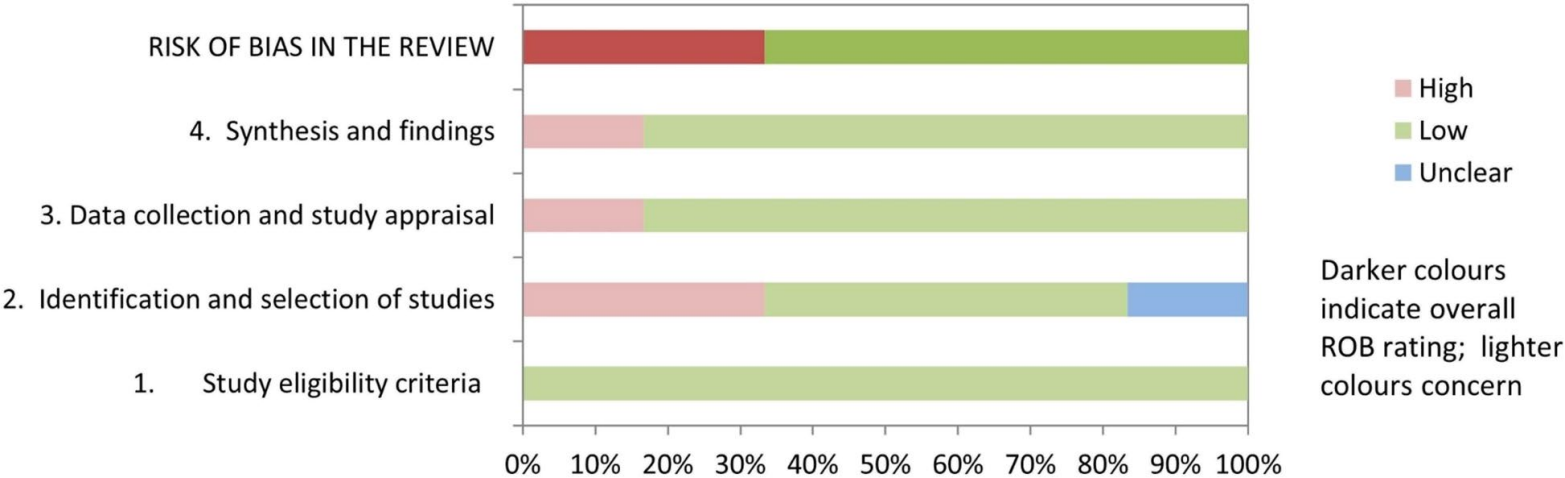
Risk of bias summary of the systematic reviews and meta-analysis included in the study based on the ROBIS results

**Fig. 3:**
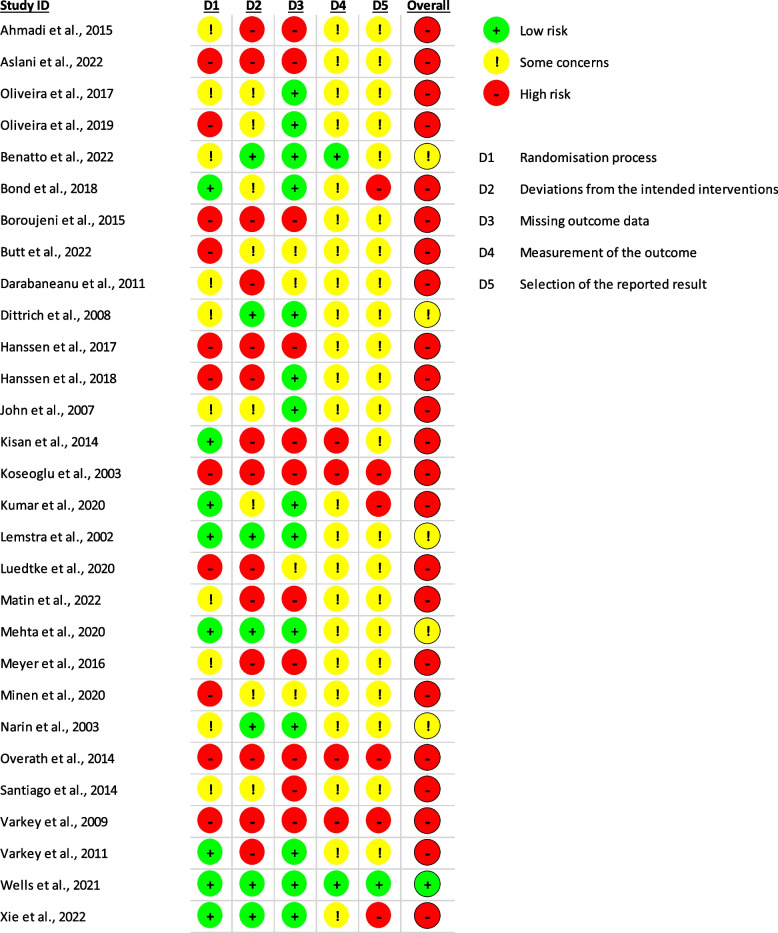
Clinical trial risk of bias assessment within studies based on the RoB 2.0 tool

**Fig. 4:**
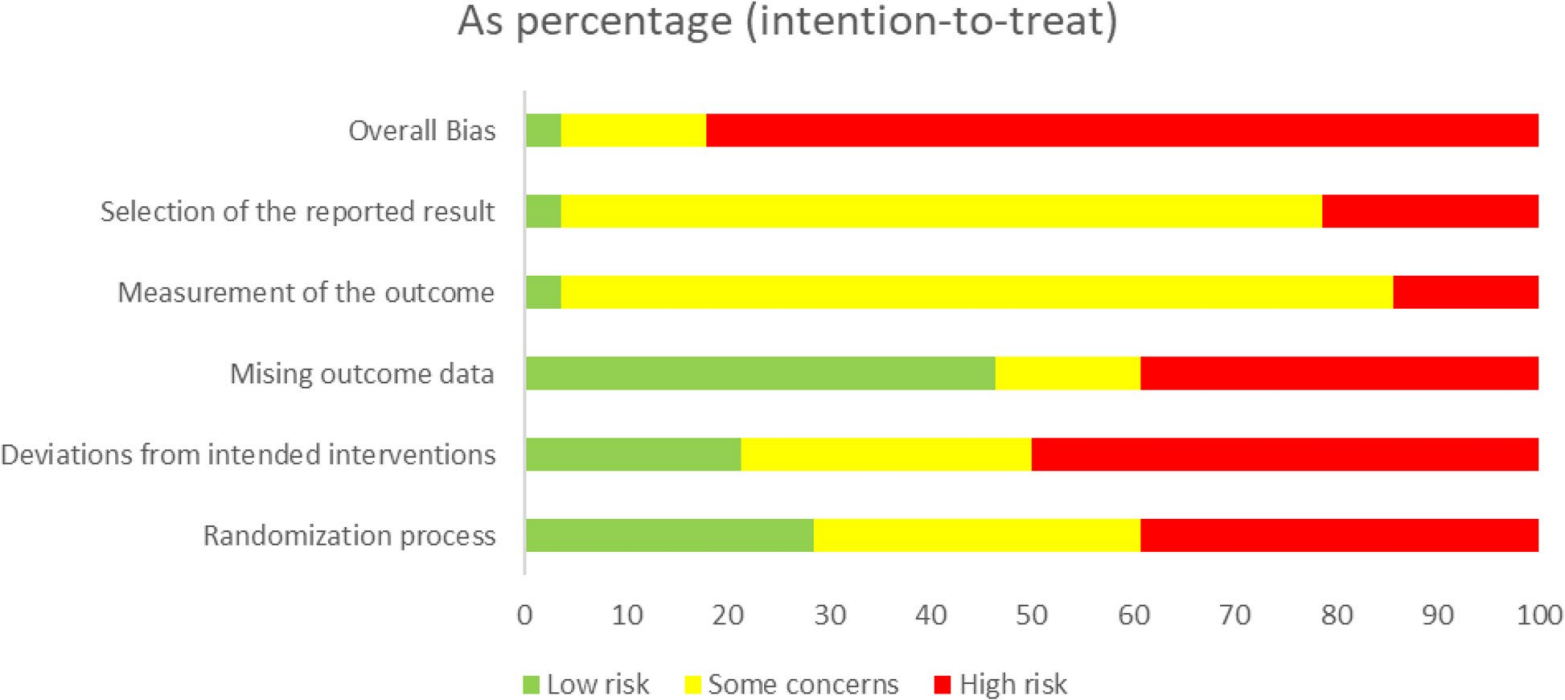
Risk of bias summary of the clinical trials included in the study based on the RoB 2.0 results

A total of 60 studies were included in the clinical practice guidelines. Our article search strategy and selection process are shown in the flow diagram (Fig. 1). The included studies were 1 umbrella review and meta-meta-analysis [35], 6 systematic reviews and meta-analyses [13, 36–40], 29 randomized controlled trials [41–69], 4 cohorts [70–73], 1 case series [74], and 19 narrative reviews [75–93]. The methodological quality and risk of bias assessment for each study are shown in Tables 2, 3, 4, 5, 6, and 7, and in Figs. 2, 3, and 4. The agreement between the evaluators in the quality assessment of the studies was high in the PEDro (κ = 0.857), RoB 2.0 (κ = 0.708), and NIH (κ = 1.000) scales, and moderate for the NOS (κ = 0.692) and SANRA scales (κ = 0.681).

**Table 2:** Modified Quality Assessment Scale for Systematic Review with the information regarding each item score and the total score for each systematic review

Studies		La Touche et al., 2020 [40]	Lemmens et al., 2019 [36]	Long et al., 2022 [37]	Luedtke et al., 2016 [38]	Varangot-Reille et al., 2021 [13]	Wu et al., 2022 [39]
**Were the search methods used to find evidence (original research) on the primary question(s) stated?**	Explicitly described to allow replication	Yes	Yes	In Part	In Part	In Part	Yes
**Was the search for evidence comprehensive?**	Adequate number and range of databases	Yes	In Part	Yes	Yes	Yes	Yes
	Alternative searches	Yes	Yes	No	Yes	Yes	Yes
	Adequate range of keywords	Yes	Yes	No	Yes	Yes	No
	Non-English language	Yes	In Part	Yes	In Part	Yes	No
**Were the criteria for deciding which studies to include in the overview reported?**	Explicitly described to allow replication	Yes	Yes	In Part	Yes	Yes	Yes
	Excludes reviews that do not adequately address inclusion and exclusion criteria	Yes	Yes	No	Yes	Yes	In Part
**Was bias in the selection of articles avoided?**	Two independent reviewers	Yes	Yes	Yes	Yes	Yes	No
**Were the criteria used for assessing the quality of included studies reported?**	Explicitly described to allow replication	Yes	Yes	In Part	Yes	Yes	Yes
**Were the methods used to combine and/or compare the findings of relevant studies appropriate?**	Meta-analysis conducted on only homogenous data or limitations to homogeneity discussed	Yes	Yes	In Part	In Part	Yes	Yes
	Confidence intervals/effect sizes reported where possible	Yes	Yes	Yes	Yes	Yes	Yes
**Were conclusions made by the author(s) appropriate?**	Supported by the meta-analysis or other data analysis findings	Yes	Yes	No	Yes	Yes	Yes
	Conclusions address levels of evidence for each intervention/comparison	Yes	Yes	No	Yes	Yes	Yes
**Total**		**26**	**24**	**12**	**23**	**25**	**19**

Item 1: Explicitly described to allow replication (ie, 100% confident that you could replicate it). If explained but you can't be 100% confident of replication = in part; Item 2: Adequate number and range of databases (3 = in part, > 3 = yes); Item 3: Alternative searches such as manual searches, Web of Science, reference lists, contact of prominent authors or other sources of information (1 of these = in part, 2 or more = yes); Item 4: Adequate range of keywords (search likely to be sensitive); Item 5: Non-English language papers included in the search. Must explicitly state that no language restrictions were applied, or something of similar meaning to score yes; Item 6: Explicitly described to allow replication (unambiguous). If described but not 100% clear = in part; Item 7: Excludes reviews that do not adequately address inclusion and exclusion criteria. One of inclusion or exclusion = in part, both = yes;Item 8: Two independent reviewers; Item 9: Explicitly described to allow replication. If the described scale is not valid, and/or reliability is not reported, score = in part; Item 10: Meta-analysis conducted on only homogenous data or limitations to homogeneity discussed; Item 11: Confidence intervals/effect sizes reported where possible; Item 12: Supported by the meta-analysis or other data analysis findings (effect sizes, confidence intervals, etc.) in the review. If only significance levels relied upon = in part; Item 13: Conclusions address levels of evidence for each intervention/comparison (eg, level A-D evidence, strong–weak evidence, etc.). Score: No = 0; In part = 1; Yes = 2. Score < 20 = low quality; Score ≥ 20 = high quality

**Table 3:** PEDro scale for randomized controlled trials with the information regarding each item score and the total score for each randomized controlled trial

Studies	Random allocation	Concealed allocation	Comparability of groups at baseline	Participant blinding	Therapist blinding	Assessor blinding	Dropouts	Intention-to-treat analysis	Intergroup statistical comparison	Point measures and variability	Total
Ahmadi et al., 2015 [41]	Yes	No	Yes	No	No	No	No	No	Yes	Yes	**4**
Aslani et al., 2021 [42]	Yes	No	Yes	No	No	No	No	No	Yes	Yes	**4**
Benatto et al., 2022 [43]	Yes	Yes	No	No	No	Yes	Yes	No	Yes	Yes	**6**
Bond et al., 2018 [44]	Yes	Yes	Yes	No	No	No	No	Yes	Yes	Yes	**6**
Boroujeni et al., 2015 [45]	Yes	No	No	No	No	No	No	No	Yes	Yes	**3**
Butt et al., 2022 [46]	No	No	Yes	No	No	No	Yes	Yes	Yes	Yes	**5**
Darabaneanu et al., 2011 [47]	No	No	Yes	No	No	No	No	No	Yes	Yes	**3**
Dittrich et al., 2008 [48]	Yes	No	Yes	No	No	No	Yes	Yes	Yes	Yes	**6**
Hanssen et al., 2017 [50]	Yes	No	No	No	No	No	No	No	Yes	Yes	**3**
Hanssen et al., 2018 [49]	Yes	No	No	No	No	No	No	No	Yes	Yes	**3**
John et al., 2007 [51]	Yes	No	Yes	No	No	No	Yes	No	Yes	Yes	**5**
Kisan et al., 2014 [52]	Yes	Yes	No	No	No	No	No	No	Yes	Yes	**4**
Köseoglu et al., 2003 [53]	No	No	No	No	No	No	Yes	No	No	Yes	**2**
Kumar et al., 2020 [54]	Yes	Yes	No	No	No	No	No	Yes	Yes	Yes	**5**
Lemstra et al., 2002 [55]	Yes	Yes	Yes	No	No	No	Yes	Yes	Yes	Yes	**7**
Luedtke et al., 2020 [56]	No	No	No	No	No	No	Yes	No	Yes	Yes	**3**
Matin et al., 2022 [57]	Yes	No	No	No	No	No	Yes	No	Yes	Yes	**4**
Mehta et al., 2021 [58]	Yes	Yes	Yes	No	No	No	Yes	Yes	Yes	Yes	**7**
Meyer et al., 2016 [59]	No	No	Yes	No	No	No	No	No	Yes	Yes	**3**
Minen et al., 2020 [60]	Yes	No	Yes	No	No	No	No	No	Yes	Yes	**4**
Narin et al., 2003 [61]	No	No	Yes	No	No	No	Yes	Yes	Yes	Yes	**5**
Oliveira et al., 2017 [62]	Yes	No	Yes	No	No	No	Yes	No	Yes	Yes	**5**
Oliveira et al., 2019 [63]	Yes	No	Yes	No	No	No	Yes	Yes	Yes	Yes	**6**
Overath et al., 2014 [64]	No	No	No	Yes	No	No	Yes	No	No	Yes	**3**
Santiago et al., 2014 [65]	Yes	No	Yes	No	No	No	No	No	Yes	Yes	**4**
Varkey et al., 2009 [66]	No	No	No	No	No	No	Yes	No	No	Yes	**2**
Varkey et al., 2011 [67]	Yes	Yes	Yes	No	No	No	No	Yes	Yes	Yes	**6**
Wells et al., 2021 [68]	Yes	Yes	Yes	Yes	No	Yes	No	Yes	Yes	Yes	**8**
Xie et al., 2022 [69]	Yes	Yes	Yes	No	No	No	No	Yes	Yes	Yes	**6**

Item 1: Subjects were randomly allocated to groups (in a crossover study, subjects were randomly allocated in the order in which treatments were received); Item 2: Allocation was concealed; Item 3: The groups were similar at baseline regarding the most important prognostic indicators; Item 4: There was blinding of all subjects; There was blinding of all therapists who administered the therapy; Item 6: There was blinding of all assessors who measured at least one key outcome; Item 7: Measures of at least one key outcome were obtained from more than 85% of the subjects initially allocated to groups; Item 8: All subjects for whom outcome measures were available received the treatment or control condition as allocated, or, where this was not the case, data for at least one key outcome were analyzed by “intention to treat”; Item 9: The results of between-group statistical comparisons are reported for at least one key outcome; Item 10: The study provides both point measures and measures of variability for at least one key outcome; Score: No = 0; Yes = 1. Poor = 0–3 points; Fair = 4–5 points; Good = 6–8 points; Excellent = 9–10 points

**Table 4:** NOS scale for cohort studies with the information regarding each item score and the total score for each cohort study

	**Selection**				**Comparability**	**Outcome**			**Total**	**Methodological Quality**
Study	Representativeness of the exposed cohort	Selection of the non-exposed cohort	Ascertainment of exposure	Outcome of interest not present at start	Comparability of cohorts on the basis of the design or analysis	Assessment of outcome	Follow-up long enough for outcomes to occur	Adequacy of follow up of cohorts		
Gaul et al., 2011 [70]	-	-	★	★	-	-	★	-	3/9	Poor
Hagan et al., 2021 [71]	★	★	-	★	★★	-	-	★	6/9	Good
Seok et al., 2020 [72]	★	★	★	★	★★	-	★	★	8/9	Excellent
Woldeamanuel et al., 2016 [73]	★	★	-	★	★★	-	★	★	7/9	Good

Scores: ★ = 1 score. Poor = 0–3 stars; Fair = 4–5 stars; Good = 6–7 stars; Excellent = 8–9 stars

**Table 5:** Item Quality Assessment Tool for Case Series Studies Scale with the information regarding each item score and the total score for the case series study

Study	Was the study question or objective clearly stated?	Was the study population clearly and fully described, including a case definition?	Were the cases consecutive?	Were the subjects comparable?	Was the intervention clearly described?	Were the outcome measures clearly defined, valid, reliable, and implemented consistently across all study participants?	Was the length of follow-up adequate?	Were the statistical methods well described?	Were the results well described?	Total
Elinoff et al., 2019 [74]	1	1	1	1	1	1	1	0	1	8 (Excellent)

Scores: No = 0; Yes = 1. Poor = 0–25%; Fair = 26–50%; Good = 51–75%; Excellent = 76–100%

**Table 6:** SANRA scale for narrative reviews with the information regarding each item score and the total score for each

Studies	Justification of the article’s importance for the readership	Statement of concrete aims of formulation of questions	Description of the literature search	Referencing	Scientific reasoning	Appropriate presentation of data	Total
Agbetoy et al., 2022 [93]	0	0	1	2	0	0	**3**
Ahn et al., 2013 [75]	0	1	0	2	1	1	**5**
Amin et al., 2018 [76]	2	2	1	2	2	2	**11**
Barber et al., 2020 [77]	2	2	0	2	2	2	**10**
Becker et al., 2009 [78]	0	0	0	0	2	1	**3**
Busch V, Gaul C, Headache, 2008 [80]	1	2	1	2	2	2	**10**
Busch V, Gaul C, Schmerz, 2008 [79]	2	2	2	2	2	2	**12**
Daenen et al., 2015 [81]	1	2	0	2	1	0	**6**
Hindiyeh et al., 2013 [82]	1	1	0	1	1	1	**5**
Irby et al., 2016 [83]	2	1	0	2	2	1	**8**
Lippi et al., 2018 [84]	2	2	1	1	0	2	**8**
Mauskop et al., 2012 [85]	1	1	0	2	1	1	**6**
Meyer et al., 2018 [86]	1	1	0	2	2	2	**8**
Guarín-Duque et al., 2021 [87]	1	2	1	2	1	0	**7**
Patel et al., 2019 [88]	2	2	0	2	2	1	**9**
Robblee et al., 2019 [89]	1	2	0	2	2	1	**8**
Song et al., 2021 [90]	1	2	1	2	2	2	**10**
Tepper et al., 2015 [91]	1	2	0	1	2	2	**8**
Wells et al., 2019 [92]	0	2	1	2	2	2	**9**

Score: No = 0; In part = 1; Yes = 2. There are no established cut-offs for different grades of quality

**Table 7:** Systematic reviews and meta-analysis risk of bias assessment within studies based on the ROBIS tool

Review	Phase 2				Phase 3
	**1. Study eligibility criteria**	**2. Identification and selection of studies**	**3. Data collection and study appraisal**	**4. Synthesis and findings**	**Risk of bias in the review**
La Touche et al., 2020 [40]					
Lemmens et al., 2019 [36]					
Long et al., 2022 [37]					
Luedtke et al., 2016 [38]					
Varangot-Reille et al., 2021 [13]					
Wu et al., 2022 [39]					

= low risk;  = high risk;  = unclear risk

### Sample characteristics

A total of 2493 patients with migraine were included in the articles that compose this clinical practice guideline, 1692 in the intervention groups and 801 in the control group. From this total sample, 1699 patients presented an episodic migraine diagnosis in 28 studies [41–45, 47–54, 57–59, 61–64, 66–71, 73, 74], and 524 had a chronic migraine diagnosis in 6 studies [44, 55, 65, 70, 72, 73]. However, 270 individuals were included in studies that did not specify the number of episodic and chronic migraine diagnoses [46, 56, 60]. The mean age range of the patients included in the studies was 29 to 51 years, and the mean body mass index range was 22.04 to 35.8 kg/m^2^. Of the total sample, 2133 were women and 360 were men.

### Outcome measures

Migraine frequency was measured by attacks per month or days with migraine per month; pain intensity was evaluated with the Visual Analog Scale (VAS), the Numeric Pain Rating Scale (NPRS), the Numeric Rating Scale (NRS), or other ordinal scales (e.g., 0 = no pain; 1 = mild; 2 = moderate; 3 = severe). Pain duration was registered as average minutes per attack, the average duration of headaches in hours, hours per day, hours per month, duration of headache episodes in days or days with a migraine episode. Disability was evaluated with the Migraine Disability Assessment questionnaire (MIDAS), the Headache Impact Test-6 (HIT-6), and the Headache Disability Index (HDI), and quality of life was measured with the Migraine Specific Quality of Life Questionnaire (MSQoL), the Migraine-Specific Quality of Life Questionnaire version 2.1 (MSQv 2.1), and the Quality of Life Profile for the Chronically Ill (PLC).

## Evidence statements and recommendations

### Grade B of recommendation

#### Aerobic exercise

This modality contains general advice for aerobic exercise without a specific definition or accurate exercise prescription parameters.

It obtained a B grade of recommendation based on the results of 1 umbrella review with meta-meta-analysis [35], and 4 systematic reviews with meta-analysis [13, 36, 38, 40] (Table 8). Professionals should consider that prescription of aerobic exercise for patients with migraine is likely to decrease pain frequency, intensity, and duration, and to improve quality of life.

**Table 8:** Summary table with each exercise modality and its respective studies

Study	Design and Diagnosis	Intervention	Key Outcomes and Follow-up	Results Within Group	Results Between Group	Level of Evidence	Adverse effects
**AEROBIC EXERCISE** **GRADE OF RECOMMENDATION: B in favour of intervention**							
**Herranz-Gómez et al., 2021 **[35]	MMA, umbrella and mapping review ICHD	**Experimental group**: Aerobic exercise, manual therapy and manual therapy with therapeutic exercise **Control group:** Any type of intervention it was possible to isolate	Frequency (days/month or days/week) Pain intensity (VAS, NPRS, numeric pain index, MVK pain scale) Disability (HDI) Quality of life (HIT-6, SF-36, SF-12)	There is moderate evidence that aerobic exercise reduces pain intensity in migraine patients. The applied interventions showed a positive effect in terms of pain intensity, quality of life and frequency		1-	No adverse effects were reported
**La Touche et al.,2020 **[40]	SR of RCTs and q-RCTs MA of RCTs Migraine with or without aura diagnosed with ICHD	**Experimental group**: aerobic exercise **Control group**: Other forms of exercise, minimal education, information, maintenance of daily living activity or drugs	Frequency (days/month or days/week) Pain intensity (VAS) Duration (hours of migraine) Quality of life (HIT-6, PLC, WHO-5, MSQoL, grading the severity of chronic pain)	It was found statistically significant differences in the decrease in pain intensity, frequency and duration of migraine in the short term, and an increase in quality of life. Aerobic exercise has low to moderate evidence in migraine patients		1-	No adverse effects were reported
**Lemmens et al., 2019 **[36]	SR of RCTs and q-RCT s MA of RCTs and Q-RCT s Migraine diagnosed with the ICHD-II	**Experimental group**: Physical endurance, physical fitness, aerobic exercise and exercise therapy performed during at least 6 weeks **Control group:** no intervention, education, treatment based on medication, relaxation therapy and advice to maintain habitual daily activity	Frequency (days/month) Pain intensity (NPRS, VAS) Duration (hours/attack and hours/month)	Significant reductions in the number of migraine days after aerobic exercise treatment were found, and small to moderate reductions in attack duration and pain intensity after aerobic exercise intervention		1-	No adverse effects were reported
**Luedtke et al., 2016 **[38]	SR of RCTs and q-RCT s MA of RCTs Migraine diagnosed with IHS criteria	**Experimental group:** standard physiotherapy (exercise, manual therapy, soft-tissue techniques, or strength and endurance training) **Control group:** placebo, standard care, waiting list or other active intervention	Frequency (number of episodes or number of headache days within a defined period of time) Pain intensity (VAS) Duration (hours or days without relief)	Aerobic exercise results suggest a statistically significant reduction in the intensity, frequency and duration of migraine		1-	No adverse effects were reported
**Varangot et al., 2021 **[13]	SR and MA of RCTs ICHD and medical diagnosis	**Experimental group:** exercise training (aerobic training, strength training, yoga or aerobic and strength training) **Control group:** non-active interventions, education, relaxation, breathing or no interventions	Frequency (painful days/month) Pain intensity (VAS, NRS) Duration (hours) Disability (HDI, HIT-6, HIT, PDI) Quality of life (SF-36)	Aerobic training has a small to moderate clinical effect on pain intensity and frequency of headache episodes in migraine patients, with very low to low certainty of evidence		1-	The study of Lemstra et al. 2002 reported minor musculoskeletal pain in 20% of patients in the intervention group, which include exercise as part of the therapy
**Ahn et al., 2013 **[75]	Narrative review			There are several lines of evidence supporting the role of exercise in migraine management. Though individually these studies have some limitations, they are still altogether compelling because this view still emerges clearly from several independent lines of investigation	-	4	No adverse effects were reported
**Amin et al., 2018** [76]	Narrative review -	-	-	It seems that although exercise can trigger migraine attacks, regular exercise may have a prophylactic effect on migraine frequency. This is most likely due to an altered migraine-triggering threshold in people who exercise regularly. Frequency and intensity of exercise that is required is unclear	-	4	Exercise can minimally trigger migraine
**Barber et al., 2020 **[77]	Narrative review	**-**	-	An aerobic exercise routine alone is sufficient to reduce migraine frequency, intensity, and duration. Higher-intensity training appears to confer more benefits. The addition of exercise to a traditional preventive regimen may provide added benefits. Patients who cannot tolerate high-impact exercise may even benefit from low-impact exercises like yoga	-	4	Exercise may induce migraine
**Busch V, Gaul C, Headache, 2008 **[80]	Narrative review -	-	-	Most of the reviewed studies did not find a significant reduction of headache attacks or headache duration and only indicate a reduction of pain intensity in migraine patients due to regular exercise	-	4	It should not be forgotten that exercise can induce sport-related headaches
**Busch V, Gaul C, Schmerz, 2008 **[79]	Narrative review -	-	-	Regular endurance sports are found in many general recommendations for the treatment of migraine patients. However, the evidence on which these recommendations are based is weak	-	4	No adverse effects were reported
**Daenen et al., 2015 **[81]	Narrative review -	-	-	Aerobic exercise on a submaximal level is the best option in migraine prophylaxis	-	4	Exercise could be a migraine-triggering factor
**Guarín-Duque et al., 2021 **[87]	Narrative review -	-	-	Adults who don't tolerate migraine drugs very well may find relief in preventive therapies such as exercise	-	4	Some authors show that exercise, especially if it is at high intensity, can trigger a migraine attack
**Hindiyeh et al., 2013 **[82]	Narrative review -	-	-	There are demonstrable differences in the way migraineurs respond to aerobic exercise during their headaches and there is more than a suggestion that migraineurs do, in fact, process the changes brought on by aerobic activity differently than non-migraineurs or migraineurs when they are inter-ictal	-	4	22% of migraineurs list exercise as a trigger
**Irby et al., 2016 **[83]	Narrative review -	-	-	Regular aerobic exercise routine is recommended as a means of managing and preventing migraine. Anyway, the optimal parameters of exercise regimens for migraine are still unclear	-	4	Physical activity may not play an important role in triggering or exacerbating migraine
**Lippi et al., 2018 **[84]	Narrative review -	-	-	High-intensity exercise should be avoided in patients with a history of exercise-provoked migraine Regular moderate aerobic physical exercise (> 40 min, 3 times per week) seems effective to reduce both the severity and frequency of migraine attacks	-	4	Since exercising may sometimes worsen migraine, being engaged in physical exercise during a migraine attack must be established on an individual basis, according to the personal history of exercise-provoked migraine
**Mauskop et al., 2012 **[85]	Narrative review -	-	-	Aerobic exercise is proven to be effective in the prevention of migraine headaches	-	4	No adverse effects were reported
**Patel et al., 2019 **[88]	Narrative review -	-	-	The overall data are still insufficient to recommend aerobic exercise as a single therapy for migraine prevention because of methodological limitations	-	4	No adverse effects were reported
**Robblee et al., 2019 **[89]	Narrative review -		-	The best current recommendation for patients with migraine is to engage in graded moderate cardiorespiratory exercise, although any exercise is better than none	-	4	No adverse effects were reported
**Song et al., 2021 **[90]	Narrative review -	-	-	Regarding efficacy, side effects, and health benefits, aerobic exercise promises to be a good strategy in the preventive treatment of migraine	-	4	Exercise can trigger a migraine attack. Pain aggravation by routine physical activity has been reported by approximately 2/3 of individuals with migraine. High-intensity exercise and an insufficient warm-up period can trigger a migraine attack
**Tepper et al., 2015 **[91]	Narrative review -	-	-	Aerobic exercise combined with behavioural therapy may be useful as a complementary migraine management	-	4	No adverse effects were reported
**Wells et al., 2019 **[92]	Narrative review	Aerobic exercise	-	Aerobic exercise reduces migraine frequency, pain intensity, duration of migraine, and migraine disability. Also, yoga and tai-chi may be beneficial for migraine patients	-	4	Physical exertion can trigger migraines in some patients
**MODERATE-INTENSITY CONTINUOUS AEROBIC EXERCISE** **GRADE OF RECOMMENDATION: B in favour of intervention**							
**Ahmadi et al., 2015 **[41]	RCT ICHD-II Episodic migraine	**Experimental group:** Aerobic exercise (*n* = 15) **Control group:** Were told not to exercise (*n* = 14)	Frequency (attacks/month) Pain intensity (VAS) Duration (average minutes/attack) Post-immediate	Significant improvement in all outcomes in the experimental group. No significant change in any variable in the control group	No significant difference between groups in any outcome	1-	No adverse effects were reported
**Oliveira et al., 2017** [62]	RCT ICHD II Episodic migraine	**Experimental Group:** Aerobic exercise (*n* = 10) **Control Group:** Waiting list (*n* = 10)	Frequency (days with migraine /month)	Significant improvement in the experimental group. No significant change in the control group	Analysis not performed	1-	No adverse effects were reported
**Oliveira et al., 2019** [63]	RCT Migraine ICHD-II Episodic migraine	**Migraine aerobic** exercise group (*n* = 13) **Migraine waitlist** group (*n* = 12) **Control aerobic** exercise group (*n* = 12) **Control waiting list** group *(n* = 13)	Frequency (attacks/month and days with migraine /month) Pain intensity (0 = no pain; 1 = mild; 2 = moderate; 3 = severe) Post-immediate	Migraine exercise: Significant improvement in attacks/month, days/month. No significant change in pain intensity Migraine waitlist: No significant change in any outcome	Favours significantly migraine exercise over migraine waitlist in days with migraine No significant change in pain intensity	1-	No adverse effects were reported
**Hanssen et al., 2017 **[50]	RCT ICHD III-B Episodic migraine	**Experimental group**: HIIT group (*n* = 16) **Experimental group**: MCT group (*n* = 16) **Control Group:** Maintain habitual daily physical activity profile and received additional standard physical activity recommendations (*n* = 16)	Frequency (days/month) Post-immediate	No significant improvement in any group	Significant difference that favours HIIT versus MCT No significant difference between HIIT-Control and MCT-Control	1-	No adverse effects were reported
**Hanssen et al., 2018 **[49]	RCT Episodic migraine without aura ICHD-IIIb	**Experimental group 1**: HIIT group (*n* = 15) **Experimental group 2:** MCT group (*n* = 15) **Control Group**: maintain habitual physical activity profile (*n* = 15)	Frequency (days/month) Post-immediate	No significant improvement in any group	Significant difference that favours HIIT versus MCT No significant difference between HIIT-Control and MCT-Control	1-	No adverse effects were reported
**Varkey et al., 2011 **[67]	RCT ICHD-II Episodic migraine	**Group 1**: Relaxation group. (*n* = 30) **Group 2:** Aerobic exercise group. (*n* = 30) **Group 3:** Topiramate group (*n* = 31)	Frequency (attacks/month and days with migraine / month) Pain intensity (VAS) Quality of life (MSQoL) Post during treatment period Post during last month of treatment Post 3 months Post 6 months	Post during the treatment period: Significant reduction in attacks/month in all groups No significant changes in other outcomes in any group	Post during the treatment period: Significant difference between groups in pain intensity favours the topiramate group No significant difference between groups in attacks/month, days with migraine/month	1-	No adverse effects were reported
				Post during the last month of treatment: Significant reduction in attacks/month in all groups No significant change in other outcomes in any group	Post during the last month of treatment: No significant difference between groups in any outcome		
				Post 3 months: Significant reduction in attacks/month in all groups No significant change in other outcomes in any group	Post 3 months: No significant difference between groups in any outcome		
				Post 6 months: Significant reduction in attacks/month in all groups No significant change in other outcomes in any group	Post 6 months: No significant difference between groups in any outcome		
**Darabaneanu et al., 2011 **[47]	Q-RCT IHS Episodic migraine with or without aura	**Experimental group:** Aerobic exercise (*n* = 8) **Control group:** No exercise (*n* = 8)	Frequency (days with migraine /month) Pain intensity (NPRS) Duration (h/month) Post-immediate Follow-up 8 weeks	Significant improvement in all outcomes in the experimental group in post-immediate. No significant change in any outcome in the control group in post-immediate	Significant interaction between exercise group and frequency and an interaction effect between exercise group and intensity of migraine attacks No significant difference in duration	1-	1 person was excluded because of pain during exercise and 4 persons because of a lack of motivation to perform the training
**Luedtke et al., 2020 **[56]	Q-RCT ICHD-III Chronic or frequent episodic migraine	**Group 1:** Standard physiotherapy (manual therapy mobilization, myofascial treatment, exercise and education) (*n* = 79) **Group 2:** Aerobic exercise (*n* = 24)	Frequency (days/month) Disability (MIDAS)	Post-immediate: No significant change in any outcome measure	Post-immediate: No significant differences in any outcome measure	1-	2 patients discontinued the aerobic group because they reported an increase in headache intensity
				Post 4 weeks: No significant change in any outcome measure	Post 4 weeks: No significant differences in any outcome measure		
				Post 3 months: No significant change in any outcome measure	Post 3 months: No significant differences in any outcome measure		
**Narin et al., 2003 **[61]	Q-RCT Episodic migraine without aura. IHS	**Experimental group:** Moderate aerobic training and medical treatment. (*n* = 20) **Control group:** Medical treatment (*n* = 20)	Pain frequency (attack/month) Pain intensity (VAS) Duration (hours) Disability (PDI) Post-immediate	Significant improvements in both groups in frequency and disability	Significant differences in pain relief favour the experimental group	1-	No adverse effects were reported
**Overath et al., 2014 **[64]	Q-RCT IHS Episodic migraine with or without aura	Exercise cohort (*n* = 28)	Frequency (attacks/month) Frequency (days/month) Post-immediate	Significant improvements in all outcomes in favour of intervention	-	1-	No adverse effects were reported
**Varkey et al., 2009** [66]	Q-RCT ICHD-II Episodic migraine with or without aura	Aerobic exercise (*n* = 26)	Frequency of days (days/month) Frequency of attacks (attacks/month) Intensity (VAS) Quality of life (MSQol) Post-immediate	Significant improvements in all outcomes in favour of intervention	-	1-	One patient reported a migraine attack immediately after training 3 dropouts because of noncompliance with the treatment, and 3 dropouts because of lack of time
**Hagan et al., 2021** [71]	Cohort Episodic migraine. ICHD-III	Exercise cohort (*n* = 98)	Headache frequency (headache/month) Intensity (VAS) Duration (hours) Post-immediate	Moderate-vigorous exercise at least three times per week had fewer headache frequency, though not statistically significant. This association was significantly stronger in those who used prophylactic medication for migraines	-	2 +	No adverse effects were reported
**YOGA** **GRADE OF RECOMMENDATION: B in favour of intervention**							
**Long et al., 2022 **[37]	SR of MA of RCT ICHD-IIIb	**Experimental group:** Yoga **Control group:** Standard treatment	Frequency (Attacks/month) Pain intensity (10-point scale) Duration (hours) Disability (MIDAS and HIT-6)	-	Compared with the control group, yoga therapy could decrease pain intensity, frequency, duration and disability	1-	No adverse effects were reported
**Wu et al., 2022 **[39]	SR and MA of RCT ICHD III	**Experimental group**: Yoga therapy **Control group:** Standard medical treatment and self-care	Frequency (headaches days/month, headaches/week) Intensity (VAS or NRS) Duration (hours) Disability (HIT-6 and MIDAS)	-	Compared with the control group, yoga therapy was associated with substantially reduced headache frequency and HIT-6 score, but revealed no obvious influence on pain intensity	1-	No adverse effects were reported
**Boroujeni et al., 2015 **[45]	RCT Episodic migraine IHS	**Experimental Group:** Yoga and pharmacological intervention (*n *= 18) **Control group:** Pharmacological intervention (*n* = 14)	Frequency (Headaches/month) Intensity (VAS) Duration (days) Disability (HIT-6) Post-immediate	Experimental group: Significant improvements in frequency, intensity and disability, but not in duration Control group: No significant improvements	Significant improvements in intensity, frequency and disability favour the experimental group. No significant differences in duration	1-	No adverse effects were reported related to yoga
**John et al., 2007 **[51]	RCT Episodic migraine without aura. IHS 2004	**Experimental group**: yoga (*n* = 36) **Control group:** self-care (*n* = 36)	Frequency (headache days/week) Intensity (NRS and VAS) Duration (hours) Post-immediate	Experimental group: Significant improvements in frequency, intensity, and duration of attack Control group: Significant increase of symptoms in all outcomes except duration	Significant improvements in frequency, intensity and duration of pain favours the experimental group	1-	No adverse effects were reported
**Kisan et al., 2014** [52]	RCT Episodic migraine ICHD-II	**Experimental group**: Yoga and conventional care (*n* = 30) **Control group:** Conventional care (*n* = 30)	Frequency (Number of headaches/month) Intensity (VAS) Disability (HIT-6) Post-immediate	Significant improvements in all outcomes in both groups	Significant improvements in all outcomes favour the experimental group in post-immediate follow-up	1-	No adverse effects were reported
**Kumar et al., 2020 **[54]	RCT Episodic migraine ICHD-III-beta	**Experimental group**: Yoga and medical therapy (n = 80) **Control group:** Medical therapy (*n* = 80)	Frequency (headaches days/month) Intensity (VAS) Disability (HIT-6 and MIDAS) Post-immediate (3 months)	Significant improvement in all outcomes in both groups	Significant improvements in all outcomes favour experimental group in post-immediate follow-up	1-	1 patient reported weight gain in the intervention group, due to medication
**Mehta et al., 2021** [58]	RCT ICHD III Episodic migraine, with or without aura	**Group 1:** Physical therapy: PMR exercise, stretching, isometric exercise of neck muscles, and cardiorespiratory endurance training. (*n* = 20) **Group 2:** Yoga. (*n* = 20) **Group 3:** Standard treatment. (*n* = 21)	Frequency (headaches/month) Intensity (VAS) Disability (HIT-6) 1 month since the initiation of the intervention 2 months since the initiation of the intervention 3 months since the initiation of the intervention (post-immediate)	Frequency: Significant reduction in all groups at 1 month, 2 month and 3 months Intensity: Significant reduction in all groups at 1 month, 2 month and 3 months Disability: Significant reduction in all groups at 2 and 3 months	Frequency reduced significantly in group 1, compared to yoga and standard treatment. No significant differences in other outcomes were observed	1-	No adverse effects were reported
**Wells et al., 2021** [68]	RCT ICHD-II Episodic migraine	**Experimental group:** Standardized training in mindfulness/yoga (*n* = 45) **Control group:** Headache education group (*n* = 44)	Frequency (migraine days/month) Intensity (VAS) Duration (no data) Disability (MIDAS and HIT-6) Quality of life (MSQv 2.1) 4 weeks post-treatment 16 weeks post-treatment 28 weeks post-treatment	At 4 weeks post-treatment, both groups showed a reduction in frequency At 4, 16 and 28 weeks post-treatment a reduction in disability and an increase in quality of life was observed in the experimental group compared with the baseline No significant changes over time in intensity and duration	Significant differences favour the experimental group in disability and quality of life at 4, 16 and 28 weeks post-treatment	1-	No adverse effects were reported due to the intervention
**Barber et al., 2020 **[77]	Narrative review	**-**	-	The addition of exercise to a traditional preventive regimen may provide added benefits. Patients who cannot tolerate high-impact exercise may even benefit from low-impact exercise like yoga	-	4	Exercise may induce migraine
**Wells et al., 2019 **[92]	Narrative review	**-**	-	Aerobic exercise reduces migraine frequency, pain intensity, duration of migraine, and migraine disability. Also, yoga and tai-chi may be beneficial for migraine patients	-	4	Physical exertion can trigger migraines in some patients
**EXERCISE AND LIFESTYLE RECOMMENDATIONS** **GRADE OF RECOMMENDATION: B in favour of intervention**							
**Lemstra et al., 2002 **[55]	RCT Chronic migraine with or without aura diagnosed with IHS criteria	**Experimental group**: exercise therapy, relaxation, stress management, massage therapy and dietary lecture. (*n* = 44) **Control group:** waiting list with standard care with patient´s family physician (*n* = 36)	Frequency (days/month) Pain intensity (VAS) Duration (hours/month) Quality of life (PDI) Post-immediate 3 months follow up	The intervention group experienced statistically significant changes in frequency, pain intensity, duration, disability and quality of life at 3 months follow-up, but not in the control group	Significant differences in frequency, intensity, duration and quality of life favour the experimental group	1-	Eight subjects in the intervention group reported minor musculoskeletal pain
**Bond et al., 2018 **[44]	RCT ICHD-III Episodic and chronic migraine with or without aura	**Experimental group**: fat-restricted diet, 250 min/week of home-based exercise and behavioural modification strategies. (*n* = 54) **Control group:** Migraine education. (n = 56)	Frequency (days/month) Pain intensity (NPRS) Duration (hours/attack) Disability (HIT-6) Post-immediate 4 months follow-up	Significant reduction in all outcomes in the control group, significant reduction in attack duration and disability but no significant change in frequency and pain intensity in the experimental group in post-immediate Significant reduction in all outcomes in the experimental and control groups except for pain intensity in the control group at follow-up	No significant difference between groups in any outcome at any endpoint assessment	1-	No adverse effects were reported
**Seok et al., 2006 **[72]	Cohort ICHD II Chronic migraine	Lifestyle recommendations with exercise cohort (*n* = 136)	Frequency (headaches/month) 1-year follow-up	Regular exercise was significant positive contributor to the reversion of transformed migraine into episodic migraine	-	2 +	No adverse effects were reported
**Woldeamanuel et al., 2016 **[73]	Cohort ICHD-IIb Chronic and episodic migraine	**Group 1**: Episodic migraine. (*n* = 175) **Group 2:** Chronic migraine (*n* = 175)	Regular lifestyle behaviours of sleep, exercise, mealtime patterns and hydration status	The chronic migraine cohort showed less regular lifestyle behaviours, including exercise habit, than the episodic migraine cohort	-	2 +	No adverse effects were reported
**Gaul et al., 2011 **[70]	Cohort ICHD-II Episodic and chronic migraine with or without aura	Muscular progressive relaxation, headache education, aerobic exercise, individual psychology therapy, group behavioural treatment with lifestyle recommendations cohort (*n* = 210)	Frequency (attacks/month and days with migraine/month)	There was a reduction of 45% in the number of attacks per month, and a mean reduction of 4 days with migraine per month Significant amount difference in adherence to lifestyle modification recommendations between the patients who showed a reduction of ≥ 50% in headache days per month and the ones who did not fulfil this outcome at the primary endpoint	-	2-	No adverse effects were reported
**Agbetou et al., 2022 **[93]	Narrative review Chronic and episodic migraine	-	-	Lifestyle modifications are essential in reducing the frequency and severity of migraine attacks. Managing obesity, alcohol, and tobacco consumption discontinuation, regular physical activity, sufficient hydration, and a healthy lifestyle are highly accessible and cost-efficient interventions for any patient with migraine	-	4	
**RELAXATION TECHNIQUES** **GRADE OF RECOMMENDATION: C in favour of intervention**							
**Meyer et al., 2016 **[59]	RCT Episodic migraine with and without aura. IHS criteria	**Group 1:** PMR training in migraine patients (*n* = 16) **Group 2:** waiting list for migraine patients (*n* = 19) **Group 3:** PMR training in healthy subjects (*n* = 21) **Group 4**: Waiting list for healthy subjects (*n* = 25)	Frequency (days/month and attacks/month) Post-immediate and follow-up of 3 months	Significant improvements in frequency in favour of PMR training in migraine group in post-immediate and follow-up	Post-immediate and follow–up: Significant differences in frequency favour the PMR training in migraine group versus the waiting list for migraine patients’ group	1-	No adverse effects were reported
**Minen et al., 2020 **[60]	RCT Episodic and chronic migraine ICHD-IIIb	**Experimental group**: PMR with a smartphone. (*n* = 77) **Control group**: only download the smartphone app. (*n* = 62)	Frequency (Days/month) Disability (MIDAS) Post-immediate Follow-up 3 months	There were no significant differences in all outcomes post-immediate and in follow-up	There was a greater no significant decline in disability in favour of the experimental group at post-immediate and follow-up	1-	No adverse effects were reported
**Varkey et al., 2011 **[67]	RCT ICHD-II Episodic migraine	**Group 1**: Relaxation group. (*n* = 30) **Group 2:** Aerobic exercise group. (*n* = 30) **Group 3:** Topiramate group (*n* = 31)	Frequency (attacks/month and days with migraine / month) Pain intensity (VAS) Quality of life (MSQoL) Post during treatment period Post during last month of treatment Post 3 months Post 6 months	Post during the treatment period: Significant reduction in attacks/month in all groups No significant changes in other outcomes in any group	Post during the treatment period: Significant difference between groups in pain intensity favours the topiramate group No significant difference between groups in attacks/month, days with migraine/month	1-	No adverse effects were reported
				Post during the last month of treatment: Significant reduction in attacks/month in all groups No significant change in other outcomes in any group	Post during the last month of treatment: No significant difference between groups in any outcome		
				Post 3 months: Significant reduction in attacks/month in all groups No significant change in other outcomes in any group	Post 3 months: No significant difference between groups in any outcome		
				Post 6 months: Significant reduction in attacks/month in all groups No significant change in other outcomes in any group	Post 6 months: No significant difference between groups in any outcome		
**Meyer et al., 2018 **[86]	Narrative review -	-	-	PMR is useful in prophylactic migraine therapy and provides indications of a cortical mechanism of action	-	4	No adverse effects were reported
**HIGH-INTENSITY AEROBIC INTERVAL TRAINING** **GRADE OF RECOMMENDATION: C in favour of intervention**							
**Hanssen et al., 2017 **[50]	RCT ICHD III-B Episodic migraine	**Experimental group**: HIIT group (*n* = 16) **Experimental group**: MCT group (*n* = 16) **Control Group:** Maintain habitual daily physical activity profile and received additional standard physical activity recommendations (*n* = 16)	Frequency (days/month) Post-immediate	No significant improvement in any group	Significant difference that favours HIIT versus MCT No significant difference between HIIT-Control and MCT-Control	1-	No adverse effects were reported
**Hanssen et al., 2018 **[49]	RCT Episodic migraine without aura ICHD-IIIb	**Experimental group 1**: HIIT group (*n* = 15) **Experimental group 2:** MCT group (*n* = 15) **Control Group**: maintain habitual physical activity profile (*n* = 15)	Frequency (days/month) Post-immediate	No significant improvement in all groups	Significant difference that favours HIIT versus MCT No significant difference between HIIT-Control and MCT-Control	1-	No adverse effects were reported
**Matin et al., 2022 **[57]	RCT ICHD II Episodic migraine	**Group 1:** HIIT (*n* = 15) **Group 2:** Supplementation (Magnesium + B12) (*n* = 15) **Group 3:** HIIT + Supplementation (*n *= 15) **Group 4:** Control group: Migraine cases (*n* = 15)	Frequency (days/month) Intensity (10/15 disabling, 5/9 moderate, ¼ mild) Duration of attacks (minutes) Disability (MIDAS) Post-immediate	Significant improvement in all outcomes in all groups	Significant improvement in all outcomes in favour of HIIT vs control	1-	No adverse effects were reported
**LOW-INTENSITY AEROBIC EXERCISE** **GRADE OF RECOMMENDATION: C in favour of intervention**							
**Santiago et al., 2014 **[65]	RCT ICHD-II Chronic migraine	**Experimental group:** Amitriptyline and aerobic exercise. (*n* = 30) **Control group**: Amitriptyline alone. (*n* = 30)	Frequency (days/month) Intensity: 1 (mild), 2 (moderate) and 3 (disabling) Duration of headache (hours/day) Post-immediate	-	Significant improvements-favour the experimental group in frequency, moderate pain intensity and duration	1-	6 persons withdrew for non-adherence to the proposed physical treatment
**Köseoglu et al., 2003 **[53]	q-RCT IHS Episodic migraine without aura	Aerobic exercise (*n* = 40)	Frequency (attacks/month) Intensity (a four-degree scale) Duration (hours of attack/month) Post-immediate	Significant improvements in all outcomes	-	1-	No adverse effects were reported
**EXERCISE AND RELAXATION TECHNIQUES** **GRADE OF RECOMMENDATION: C in favour of intervention**							
**Dittrich et al., 2008 **[48]	RCT Episodic migraine with and without aura ICHD-I	**Experimental group:** Aerobic exercise group and relaxation (n = 15) **Control group:** information about Physical activity (n = 15)	Frequency (attacks/month) Pain intensity (slight, moderate, intense, very intense, intolerable) Quality of life (PLC)	There were no significant differences in any outcome except in pain intensity in favour of the exercise group at post-immediate	There were no significant differences in any outcome at post-immediate	1-	No adverse effects were reported
**Mehta et al., 2021** [58]	RCT ICHD III Episodic migraine, with or without aura	**Group 1:** Physical therapy: PMR exercise, stretching, isometric exercise of neck muscles, and cardiorespiratory endurance training. (n = 20) **Group 2:** Yoga. (n = 20) **Group 3:** Standard treatment. (n = 21)	Frequency (headaches/month) Intensity (VAS) Disability (HIT-6) 1 month since the initiation of the intervention 2 months since the initiation of the intervention 3 months since the initiation of the intervention (post-immediate)	Frequency: Significant reduction in all groups at 1 month, 2 month and 3 months Intensity: Significant reduction in all groups at 1 month, 2 month and 3 months Disability: Significant reduction in all groups at 2 and 3 months	Frequency reduced significantly in group 1, compared to yoga and standard treatment. No significant differences in other outcomes were observed	1-	No adverse effects were reported
**Butt et al., 2022 **[46]	Q-RCT Episodic and chronic migraine	**Experimental group**: supervised exercises protocol, including aerobic exercise and PMR (n = 14) **Control group:** prophylactic medicines (n = 14)	Pain Intensity (NPRS) Disability (MIDAS, HIT-6, HDI) Post-immediate	There were significant differences in all outcomes in both groups at post-immediate	There were significant differences in all outcomes between groups at post-immediate that favour the experimental group	1-	No adverse effects were reported
**Becker et al., 2009 **[78]	Narrative review -	Multidisciplinary treatment, not only medication management is needed in migraine patients. Exercise and relaxation techniques are important components of stress and symptomatic management. For migraine, a more substantial relaxation training program might be necessary	-	-	-	4	No adverse effects were reported
**NECK STRENGTH EXERCISE** **GRADE OF RECOMMENDATION: C against the intervention**							
**Benatto et al., 2022 **[43]	RCT Episodic migraine ICHD-III	**Experimental group:** craniocervical muscle-strengthening exercise (*n* = 21) **Control group:** sham ultrasound group (*n* = 21)	Frequency (days with headache/month) Intensity (NRS) Disability (MIDAS) Post-immediate 1-month post-intervention 2-month post-intervention 3-month post-intervention	Only significant difference in the intensity of headache for both groups	No significant differences in any outcome	1-	No adverse effects were reported
**TAI-CHI** **GRADE OF RECOMMENDATION: C in favour of intervention**							
**Xie et al., 2022 **[69]	RCT ICHD-III Episodic migraine	Experimental group: Tai Chi (*n* = 40) Control group: Waiting list (*n* = 33)	Frequency (attacks/month and days with migraine/month) Intensity (VAS) Duration (hours/attack)	Significant reduction in migraine in frequency (both attacks and days with migraine per month) intensity and duration in Tai Chi group at the end of treatment and follow-up Participants in waiting list only found significant reduction in days with migraine at follow-up	Significant reduction in Tai Chi group compared to control group only in frequency (both attacks and days with migraine per month) at the end of treatment and follow-up No significant differences in intensity or duration	1-	Joint pain (33.8%), muscle pain (33.3%), slight sprain (10.2%) and dizziness (5.1%) All participants indicated tolerability of these symptoms. No serious cases appeared
**Wells et al., 2019 **[92]	Narrative review	-	-	Aerobic exercise reduces migraine frequency, pain intensity, duration of migraine, and migraine disability. Also, yoga and tai-chi may be beneficial for migraine patients	-	4	Physical exertion can trigger migraines in some patients
**RESISTANCE EXERCISE** **GRADE OF RECOMMENDATION: C in favour of intervention**							
**Aslani et al., 2021** [42]	RCT Episodic migraine ICHD	**Experimental group**: Resistance training. (*n* = 10) **Control group**: No exercise. (*n* = 10)	Frequency (attacks/month) Intensity (VAS) Duration (Days) Quality of life (HIT-6) Post-immediate	All outcomes improved significantly in the exercise group in the pre-post measures	There were significant differences that favour resistance training in all outcomes	1-	No adverse effects were reported
**QI-GONG** **GRADE OF RECOMMENDATION: D in favour of intervention**							
**Elinoff et al., 2019** [74]	Case series ICHD-II Episodic migraine	Kiko Exercise and its background (*n* = 13)	Frequency (attack/month) Intensity (1 to 5 scale) Disability (MIDAS) Post-immediate	Disability score reduced by 50% in 4/6 patients Intensity did not show improvement Frequency was improved in more than 1 attack in 3/6 patients	-	3	No adverse effects were reported

*Abbreviations: HDI* Headache Disability Index, *HIIT* High-Intensity Interval Training, *ICHD* International Classification of Headache Disorders, *HIT* Headache Impact Test, *HIT-6* Headache Impact Test-6. *HIS* International Headache Society, *MA* Meta-Analysis, *MCT* Moderate Continuous Training, *MIDAS* Migraine Disability Assessment questionnaire, *MMA* Meta-Meta-Analysis, *MSQoL* Migraine Specific Quality of life Questionnaire, *MSQv 2.1* Migraine-Specific Quality of Life Questionnaire version 2.1, *MVK pain scale* Modified Von Korff pain scale, *NPRS* Numeric Pain Rating Scale, *NRS* Numeric Rating Scale, *PDI* Pain Disability Index, *PLC* Quality of Life Profile for the Chronically Ill, *PMR* Progressive Muscle Relaxation, *q-RCT* Quasi-Randomized Clinical Trial, *RCT* Randomized Controlled Trial, *SF-12* Short Form-12 Health Survey, *SF-36* Short Form-36 Health Survey, *SR* Systematic Review, *VAS* Visual Analogue Scale, *WHO-5* Five Well-Being Index

#### Moderate-intensity continuous aerobic exercise

This modality is defined as an exercise intervention that uses large muscle groups, with increased breathing and continuously maintaining a heart rate at an intensity from 12–16 on the Borg perceived exertion scale, a 64%-76% estimated maximum heart rate (HRmax), a 40%-59% heart rate reserve (HRR), or a 40%-59% oxygen uptake reserve (VO_2_R) [95].

It reached a B grade of recommendation based on the results of 6 randomized controlled trials [41, 49, 50, 62, 63, 67], 5 quasi-randomized controlled trials [47, 56, 61, 64, 66], and 1 cohort study [71] (Table 8). A total of 564 participants were included in these studies, of whom 436 were diagnosed with episodic migraine, 103 were not clearly differentiated between episodic or chronic migraine diagnoses, and 25 were healthy controls.

Professionals should consider that moderate-intensity continuous aerobic exercise, from an 8-week onward intervention applied 3 times per week, is likely to improve headache frequency, might improve pain intensity, and remotely improves attack duration, disability and quality of life in patients with episodic migraine (Table 9).

**Table 9:** Highlighted phrases to summarize the strength of recommendation for each exercise modality

Intervention	Migraine diagnosis	Effect	Grade of recommendation	Studies	Outcomes	Results
**Moderate-intensity continuous aerobic exercise**	Episodic migraine	Moderate-intensity continuous aerobic exercise, from an 8-week onward intervention applied 3 times per week is likely to improve headache frequency, might improve pain intensity, and remotely improve attack duration, disability, and quality of life in patients with episodic migraine	B in favor of intervention	*N* = 12 RCTs (*n* = 6): Hanssen 2018 [49], Varkey 2011 [67], Hanssen 2017 [50], Oliveira 2017 [62], Oliveira 2019 [63] , Ahmadi 2015 [41] q-RCTs (*n* = 5): Darabaneanu 2011 [47], Luedtke 2020 [56], Varkey 2009 [66], Overath 2014 [64], Narin 2003 [61] Cohort (*n* = 1): Hagan 2021 [71]	**Frequency** (*n* = 12): Hanssen 2018 [49], Varkey 2011 [67] , Hanssen 2017 [50], Oliveira 2017 [62], Oliveira 2019 [63], Ahmadi 2015[41], Darabaneanu 2011 [47], Varkey 2009[66], Overath 2014 [64], Luedtke 2020 [56], Narin 2003 [61], Hagan 2021 [71]	**Positive effect** (*n* = 8): Varkey 2011 [67], Oliveira 2017 [62], Oliveira 2019 [63], Ahmadi et 2015 [47], Darabaneanu 2011 [47], Varkey 2009 [66], Overath 2014 [64], Narin 2003 [61] **Without effect** (*n* = 4): Hanssen 2018 [49], Hanssen 2017 [50], Luedtke 2020 [56], Hagan 2021 [71]
					**Pain intensity** (*n* = 7): Oliveira 2019 [63], Ahmadi 2015 [41], Varkey 2011 [67], Darabaneanu 2011 [47], Varkey 2009 [66], Narin 2003 [61], Hagan 2021 [71]	**Positive effect** (*n *= 4): Ahmadi 2015 [41], Darabaneanu 2011 [47], Varkey 2009 [66], Narin 2003 [61] **Without effect** (*n* = 3): Oliveira 2019 [63], Varkey 2011 [67], Hagan 2021 [71]
					**Duration** (*n* = 4): Ahmadi 2015 [41], Darabaneanu 2011 [47], Narin 2003 [61], Hagan 2021 [71]	**Positive effect** (*n* = 1): Ahmadi 2015 [41] **Without effect** (*n* = 3): Darabaneanu 2011 [47], Narin 2003 [61], Hagan 2021 [71]
					**Disability** (*n* = 2): Luedtke 2020 [56], Narin 2003 [61]	**Positive effect** (*n* = 1): Narin 2003 [61] **Without effect** (*n* = 1): Luedtke 2020 [56]
					**Quality of life** (*n* = 3): Varkey 2011 [67], Varkey 2009 [66], Narin 2003 [61]	**Positive effect** (*n* = 1): Varkey 2009 [66] **Without effect** (*n* = 2): Varkey 2011 [67], Narin 2003 [61]
**Yoga**	Episodic migraine	Yoga, including asanas, breathing and relaxation techniques, and meditation is likely to improve headache frequency and disability and remotely improves pain intensity and attack duration, from a 6-week onward intervention applied 3 times per week in episodic migraine	B in favor of intervention	*N* = 8 SR and MA in RCT (*n* = 2): Wu 2022 [39], Long 2022 [37] RCT (*n* = 6): Kumar 2020 [54], Kisan 2014 [52], Boroujeni 2015 [45], John 2007 [51], Mehta 2021 [58], Wells 2021 [68]	**Frequency** (*n* = 8): Wu 2022 [39], Kumar 2020 [54], Kisan 2014 [52], Boroujeni 2015 [45], John 2007 [51], Mehta 2021 [58], Wells 2021 [68], Long 2022 [37]	**Positive effect** (*n* = 8): Wu 2022 [39], Kumar 2020 [54], Kisan 2014 [52], Boroujeni 2015 [45], John 2007 [51], Mehta 2021 [58], Wells 2021 [68], Long 2022[37]
					**Pain intensity** (*n* = 8): Wu 2022 [39], Kumar 2020 [54], Kisan 2014 [52], Boroujeni 2015 [45], John 2007 [51], Mehta 2021 [58], Wells 2021 [68], Long 2022 [37]	**Positive effect** (*n* = 4): Kumar 2020 [54], Kisan 2014 [52], John 2007 [51], Long 2022 [37] **Without effect** (*n* = 4): Wu 2022 [39], Mehta 2021 [58], Boroujeni 2015 [45], Wells 2021 [68]
					**Disability** (*n* = 7): Wu 2022 [39], Kumar 2020 [54], Kisan 2014 [52], Boroujeni 2015 [45], Mehta 2021 [58], Wells 2021 [68], Long 2022 [37]	**Positive effects** (*n* = 6) Wu 2022 [39], Kumar 2020 [54], Kisan 2014 [52], Boroujeni 2015 [45], Wells 2021 [68], Long 2022 [37]
						**Without effects** (*n* = 1): Mehta 2021 [58]
					**Duration** (*n* = 6): Wu 2022, Boroujeni 2015 [45], John 2007 [51], Mehta 2021 [58], Wells 2021 [68], Long 2022 [37]	**Positive effect** (*n* = 3): John 2007 [51], Mehta 2021 [58], Long 2022 [37] **Without effect** (*n* = 3): Wu 2022 [39] , Boroujeni 2015 [45], Wells 2021 [68]
**Exercise and lifestyle recommendations**	Episodic and chronic migraine	Exercise prescription and physical activity in conjunction with other lifestyle recommendations is likely to decrease pain frequency, might improve pain intensity and duration, and remotely decrease disability of patients with episodic and chronic migraine after 6 weeks of intervention with 3–5 sessions per week. Moreover, it remotely improve the function and quality of life of patients with chronic migraine	B in favor of intervention	*N* = 5 RCT (*n* = 2): Bond 2018 [44], Lemstra 2002 [55] Cohort (*n* = 3): Seok 2006 [72], Woldeamanuel 2016 [73] Gaul 2011 [70]	**Frequency** (*n* = 5): Bond 2018 [44], Lemstra 2002 [55], Seok 2006 [72], Woldeamanuel 2016 [73], Gaul 2011 [70]	**Positive effect** (*n* = 5): Bond 2018 [44], Lemstra 2002 [55], Seok 2006 [72], Woldeamanuel 2016 [73], Gaul 2011 [70]
					**Pain intensity** (*n* = 2): Bond 2018 [44], Lemstra 2002 [55]	**Positive effect** (*n* = 2): Bond 2018 [44], Lemstra 2002 [55]
					**Duration** (*n* = 2): Bond 2018 [44], Lemstra 2002 [55]	**Positive effect** (*n* = 2): Lemstra 2002 [55], Bond 2018 [44]
					**Disability** (*n* = 1): Bond 2018 [44]	**Positive effect** (*n* = 1): Bond 2018 [44]
					**Quality of life** (*n* = 1): Lemstra 2002 [55]	**Positive effect** (*n* = 1): Lemstra 2002 [55]
**Relaxation** **Techniques**	Episodic and chronic migraine	Relaxation techniques remotely improve headache frequency after at least 6 weeks, from 1 session per week to daily sessions, in patients with episodic but not chronic migraine. It remotely improves pain intensity after 12 weeks of intervention with 3 sessions per week in patients with episodic migraine	C in favor of intervention	*N* = 3 RCT (*n* = 3): Meyer 2016 [59], Minen 2020 [60], Varkey 2011 [67]	**Frequency** (*n* = 3): Meyer 2016 [59], Minen 2020 [60], Varkey 2011 [67]	**Positive effect** (*n* = 2): Meyer 2016 [59], Varkey 2011 [67] **Without effect** (*n* = 1): Minen 2020 [60]
					**Pain Intensity** (*n* = 1): Varkey 2011 [67]	**Positive effect** (*n* = 1): Varkey 2011 [67]
					**Disability** (*n* = 1): Minen 2020 [60]	**Without effect** (*n* = 1): Minen 2020 [60]
					**Quality of life** (*n* = 1): Varkey 2011 [67]	**Without effect** (*n* = 1): Varkey 2011 [67]
**High-intensity aerobic interval training**	Episodic migraine	High-intensity aerobic interval training might improve the frequency of pain and remotely improve the intensity of pain, duration, and disability after 8 weeks of intervention with 3 sessions per week in patients with episodic migraine	C in favor of intervention	*N* = 3 RCT (*n* = 3): Hanssen 2017 [50], Hanssen 2018 [49], Matin 2022 [57]	**Frequency** (*n* = 3): Hanssen 2017 [50], Hanssen 2018 [49], Matin 2022 [57]	**Positive effect** (*n* = 3) Hanssen 2017 [50], Hanssen 2018 [49], Matin 2022 [57]
					**Intensity of pain** (*n* = 1): Matin 2022 [57]	**Positive effect** (*n* = 1) Matin 2022 [57]
					**Duration** (*n* = 1): Matin 2022 [57]	**Positive effect** (*n* = 1) Matin 2022 [57]
					**Disability** (*n* = 1): Matin 2022 [57]	**Positive effect** (*n* = 1) Matin 2022 [57]
**Low-intensity aerobic exercise**	Episodic migraine	Low-intensity aerobic exercise remotely improves headache frequency, intensity of pain, and total duration per month of migraine, after 6 weeks of intervention with 3 sessions per week in patients with episodic migraine	C in favor of intervention	*N* = 2 RCT: Santiago 2014 [65] q-RCT: Köseoglu [53]	**Frequency, intensity, and duration** (*n* = 2): Köseoglu [53] Santiago 2014 [65]	**Positive effect in all outcomes** (*n* = 2): Köseoglu [53] Santiago 2014 [65]
**Exercise and relaxation techniques**	Episodic and chronic migraine	Exercise and relaxation techniques might improve pain intensity and remotely improve frequency and disability of patients with episodic and chronic migraine after 6 weeks of intervention with at least 2 days per week of sessions	C in favour of intervention	*N* = 3 RCT (*n* = 2): Dittrich 2008 [48], Mehta 2021 [58] q-RCT (*n* = 1): Butt 2022 [46]	**Frequency** (*n* = 2): Dittrich 2008 [48], Mehta 2021 [58]	**Positive effect** (*n* = 1): Mehta 2021 [58] **Without effect** (*n* = 1): Dittrich 2008 [48]
					**Pain intensity** (*n* = 3): Butt 2022 [46], Dittrich 2008 [48], Mehta 2021 [58]	**Positive effect** (*n* = 3): Butt 2022 [46], Dittrich 2008 [48], Mehta 2021 [58]
					**Disability** (*n* = 2): Butt 2022 [46], Mehta 2021 [58]	**Positive effect** (*n* = 2)**:** Butt 2022 [46], Mehta 2021 [58]
					**Quality of life** (*n* = 1): Dittrich 2008 [48]	**Without effect** (*n* = 1)**:** Dittrich 2008 [48]
**Neck strength exercise**	Episodic migraine	Neck resistance exercise might not improve migraine frequency, pain intensity, or disability of patients with episodic migraine after 8 weeks of intervention with at least 1 supervised session per week and daily home exercises done twice a day	C against the intervention	*N* = 1 RCT: Benatto 2022 [43]	**Frequency, intensity, and disability** (*n* = 1): Benatto 2022 [43]	**Without effect in all outcomes** (*n* = 1): Benatto 2022 [43]
**Tai-Chi**	Episodic migraine	Tai Chi remotely improves migraine frequency in episodic migraine patients after 12 weeks of intervention with 5 sessions per week Tai Chi might not improve pain intensity or attack duration in episodic migraine patients	C in favor of intervention	*N* = 1 RCT (*n* = 1): Xie 2022 [69]	**Frequency** (*n* = 1): Xie 2022 [69]	**Positive effect** (*n* = 1): Xie 2022 [69]
					**Pain intensity** (*n* = 1): Xie 2022 [69]	**Without effect** (*n* = 1): Xie 2022 [69]
					**Duration** (*n* = 1): Xie 2022 [69]	**Without effect** (*n* = 1): Xie 2022 [69]
**Resistance exercise**	Episodic migraine	Resistance exercise remotely improves pain frequency, intensity, and quality of life of patients with episodic migraine after 8 weeks of intervention with at least 3 sessions per week	C in favor of intervention	*N* = 1 RCT: Aslani 2021 [42]	**Frequency, intensity, and disability** (*n* = 1): Aslani [42]	**Positive effect in all outcomes** (*n* = 1): Aslani [42]
**Qi-Gong**	Episodic migraine	Qi-Gong remotely improves pain frequency and disability of patients with episodic migraine after 3 months of intervention with daily sessions. It might not improve pain intensity of patients with episodic migraine	D in favor of intervention	*N *= 1 Case series: Elinoff 2019 [74]	**Frequency and disability** (*n* = 1): Elinoff 2009 [74]	**Positive effects** (*n* = 1): Elinoff 2009 [74]
					**Pain intensity** (*n* = 1): Elinoff 2019 [74]	**Without effect** (*n* = 1): Elinoff 2019 [74]

*RCT* Randomized controlled trial, *q-RCT* Quasi-randomized controlled trial

#### Yoga

Yoga is defined as a mind–body intervention that includes 3 components: physical alignment poses (asanas), breathing techniques, and mindfulness exercises (meditations). Its intensity varies from light to vigorous and includes strength, balance, coordination, and flexibility components [96–98].

It obtained a B grade of recommendation based on the results of 2 systematic reviews with meta-analysis [37, 39], and 6 randomized controlled trials [45, 51, 52, 54, 58, 68] (Table 8). A total of 467 patients with episodic migraine were included in these studies.

Professionals should consider that yoga, including asanas, breathing and relaxation techniques, and meditation is likely to improve headache frequency and disability and remotely improves pain intensity and attack duration, from a 6-week onward intervention applied 3 times per week for episodic migraine (Table 9).

#### Exercise and lifestyle recommendations

This recommendation is defined as the conjunction of interventions directed to implement habits regarding physical activity, mealtimes, sleep, medication consumption and stress management. Some specific recommendations included here are focused to achieve regular exercise, regular sleep hours along the week, keeping consistent meal hours, adequate hydration, relaxation for stress management and avoiding excessive medication intake.

This modality achieved a B grade of recommendation based on the results of 2 randomized controlled trials [44, 55], and 3 cohorts [70, 72, 73] (Table 8). A total of 954 individuals participated in these studies, divided in 490 episodic and 464 chronic migraine patients.

Professionals should consider that exercise prescription and physical activity in conjunction with other lifestyle recommendations is likely to decrease pain frequency, might improve pain intensity and attack duration, and remotely decrease the disability of both episodic and chronic migraine patients after 6 weeks of intervention with 3–5 sessions per week. Moreover, it remotely improves the function and quality of life of patients with chronic migraine (Table 9).

### Grade C of Recommendation

#### Relaxation techniques

These are defined as techniques commonly employed for headache treatment that include progressive muscle relaxation to help patients identify and discriminate between tense and relaxed muscle groups, autogenic training or cued relaxation, visualization and guided imagery, diaphragmatic breathing, and mini-relaxation, which focuses on a limited number of muscles in the head, neck, and shoulders [99].

This modality reached a C grade of recommendation based on the results of 3 randomized controlled trials [59, 60, 67] (Table 8). A total of 311 individuals participated in these studies, of whom 126 had episodic migraine, 139 had no clear differential diagnosis between episodic or chronic migraine, and 46 were healthy controls.

Professionals should consider that relaxation techniques remotely improve headache frequency after at least 6 weeks, from 1 session per week to daily sessions, in patients with episodic migraine. It remotely improves pain intensity after 12 weeks of intervention with 3 sessions per week in patients with episodic migraine (Table 9).

#### High-intensity interval training

This modality is defined as exercise that involves alternating periods of high-intensity aerobic exercise at or below maximal oxygen uptake with light recovery exercise or no exercise between intervals [100].

It obtained a C grade of recommendation based on the results of 3 randomized controlled trials [49, 50, 57] (Table 8). A total of 133 patients with episodic migraine were included in these studies.

Professionals should consider that high-intensity aerobic interval training might improve the frequency of pain and remotely improve the intensity of pain, attack duration, and disability after 8 weeks of intervention with 3 sessions per week in patients with episodic migraine (Table 9).

#### Low-intensity continuous aerobic exercise

The definition of this modality is any activity that uses large muscle groups, increases breathing and heart rate, and can be maintained continuously and rhythmically, using aerobic metabolism to extract energy, at an intensity from 8–11 on the Borg perceived exertion scale, 50%-63% HRmax, 20%-39% HRR, or 20%-39% VO_2_R [95].

This modality achieved a C grade of recommendation based on the results of 1 randomized controlled trial [65], and 1 quasi-randomized trial [53] (Table 8). A total of 40 episodic and 60 chronic migraine patients participated in these studies.

Professionals should consider that low-intensity aerobic exercise remotely improves headache frequency, pain intensity, and total duration per month of migraine after 6 weeks of intervention with 3 sessions per week in patients with episodic migraine (Table 9).

#### Exercise and relaxation techniques

This modality consists of the combination of exercise and relaxation techniques, previously defined.

This combination of techniques reached a C grade of recommendation based on the results of 2 randomized controlled trials [48, 58], and 1 quasi-randomized trial [46] (Table 8). A total of 119 patients with migraine were included in these studies, of whom 91 were patients with episodic migraine and 28 had no clear differential diagnosis between episodic or chronic migraine.

Professionals should consider that exercise and relaxation techniques might improve pain intensity and remotely improve the frequency and disability of episodic and chronic migraine patients after 6 weeks of intervention with at least 2 days per week of sessions (Table 9).

#### Neck strength exercise

This exercise modality consists of motor control and resistance exercise directed to the deep and superficial muscles of the neck and craniocervical regions with the aim of gaining strength.

This modality achieved a C grade of recommendation against this intervention based on the results of 1 randomized controlled trial [43] (Table 8). This study included a total of 42 patients with episodic migraine.

Professionals should consider that neck resistance exercise might not improve migraine frequency, pain intensity, or disability of patients with episodic migraine after 8 weeks of intervention with at least 1 supervised session per week and daily home exercises performed twice a day (Table 9).

#### Tai chi

Tai Chi is considered a balance training program that contains slow movements that stress postural control, can be performed in groups and requires the person to move body parts gently and slowly while breathing deeply [101].

This modality obtained a C grade of recommendation based on the results of 1 randomized controlled trial [69], and 1 narrative review [92] (Table 8). The randomized controlled trial included a total of 73 patients with episodic migraine.

Professionals should consider that Tai Chi remotely improves migraine frequency in episodic migraine patients after 12 weeks of intervention with 5 sessions per week. It might not improve pain intensity or attack duration (Table 9).

#### Resistance exercise

Resistance exercise is defined as an exercise modality that provokes an improvement in functional performance by increasing muscular strength, power, speed, hypertrophy, local muscular resistance, motor performance, balance, and coordination [102].

It obtained a C grade of recommendation based on the results of 1 randomized controlled trial [42] (Table 8). A total of 20 patients with episodic migraine participated in this study.

Professionals should consider that resistance exercise remotely improves pain frequency and intensity and disability of patients with episodic migraine after 8 weeks of intervention with at least 3 sessions per week (Table 9).

### Grade D of recommendation

#### Qi-Gong

Qi-Gong is a series of exercises that incorporates elements of slow, gentle movement, and awareness and regulation of breathing, as well as the intentional direction of thoughts, attention, imagery, and sensation [103].

This modality achieved a D grade of recommendation based on the results of a case series study [74] (Table 8). Only 6 patients with episodic migraine were analyzed in this study.

Professionals should consider that Qi-Gong remotely improves pain frequency and disability of patients with episodic migraine after 3 months of intervention with daily sessions. It might not improve the pain intensity of patients with episodic migraine (Table 9).

#### Prescription exercise parameters

The prescription parameters used in each study are included in Table 10. The summary of the prescription parameters recommended for prescribing each exercise modality in patients with migraine is shown in Table 11.

**Table 10:** Prescription parameters used in each of the included studies for each exercise modality

Type of intervention	Trial	Design	Group	Distribution	Frequency	Duration	Intensity	Exercise testing
**Moderate intensity continuous aerobic exercise**	**Ahmadi et al. 2015 **[41]	RCT	Exercise Group	Supervised/Unsupervised: No info Warm-up = 15 min Main training = 20 min Cool down = 5 min Training material = No info	3 times/week for 8 weeks	Total duration = 40 min	Warm up = Gradual increase in intensity between 11–13 Borg Main training = Gradual increase in intensity between 14–16 Borg Cool down = Borg 11–13	-
	**Oliveira et al. 2017** [62]	RCT	Exercise group	Supervised exercise Warm-up = 5 min walking on a treadmill Main training = 30 min walking on a treadmill Cool-down = 5 min walking on a treadmill	3 times/week for 12 weeks	Total duration = 40 min	Main training = intensity corresponding to the participant’s ventilatory threshold	-VO_2_ max -Ventilatory threshold
	**Oliveira et al. 2019** [63]	RCT	Exercise group	Supervised exercise Warm-up = 5 min walking/jogging on a treadmill Main training = 30 min walking/jogging on a treadmill Cool-down = 5 min walking/jogging on a treadmill	3 times /week for 12 weeks	Total duration = 40 min	Main training = speed (m/min), HR, and self-perceived effort corresponding to the participant’s ventilatory threshold	- VO_2_ max -Ventilatory threshold
	**Hanssen et al. 2017 **[50]	RCT	MCT	Supervised exercise Warm-up = 400 m easy running on a treadmill and 2 skipping exercises Main training = Continuous running on a treadmill Cool down = 400 m easy running on a treadmill and stretching exercises	2 times/week for 12 weeks	Main training = 45 min	Main training = 70% HRmax (± 5 bpm)	-Individual anaerobic lactate-threshold -HRmax - VO_2_ max (supervised)
	**Hanssen et al. 2018 **[49]	RCT	MCT	Supervised exercise Warm-up = 400 m easy running on a treadmill and 2 skipping exercises Main training = Continuous running on a treadmill Cool down = 400 m easy running on a treadmill and stretching exercises	2 times/week for 12 weeks	Main training = 45 min	Main training = 70% HRmax (± 5 bpm)	-Individual anaerobic lactate-threshold -HRmax - VO_2_ max (supervised)
	**Varkey et al. 2011 **[67]	RCT	Exercise group	Supervised exercise Exercise (15 min warm-up, 20 min exercise and 5 min cool-down)	3 times/week for 12 weeks	Total duration = 40 min	Exercise group intensity based on a Borg’s scale of Rated Perceived Exertion (6–20) -Warm up: 11–13 -Exercise: 14–16 -Cool-down: 11–13	
	**Darabaneanu et al. 2011 **[47]	q-RCT	Exercise group	Supervised exercise Warm-up = 10 min on a treadmill Main training = Jogging on a treadmill Cool down = 10 min on a treadmill	3 times/week for 10 weeks	Main training jogging duration of 14^th^-30^th^ session: 30 min continuously Main training jogging-walking intervals duration from 1^st^ to 13^th^ session (min): 1–2, 2–2, 2–1, 3–1, 3–1, 4–1, 4–1, 5–1, 7–1, 7–1, 10–1, 10–1, 10–1	-	-
	**Luedtke et al. 2020 **[56]	q-RCT	Supervised Aerobic Exercise Group	Warm-up = 5–10 min Main training = 30 min aerobic exercise Cool-down = 5–10 min Supervised aerobic exercise modalities = cycling ergometer, treadmill, or cross-trainer Unsupervised aerobic exercise modalities = nordic walking, slow running, outdoor cycling, swimming, cycling ergometer, other activities	2 times/week for 5 weeks 1st session/week supervised 2^nd^ session/week unsupervised	Total duration = 40–50 min	Main training for non-trained patients:11–13 Borg Main training for trained patients:14–15 Borg	-
	**Narin et al. 2003 **[61]	q-RCT	Exercise group	Supervised exercise 5 min warm-up, 10 min cycling, 10 min walking on a treadmill, 5 min stepper, 10 min training upper extremities at the power station, 10 repetitions of neck and postural exercises, 10 repetitions of rowing and 5 min of cool-down	3 times/week for 8 weeks	Total duration = 60 min		
	**Overath et al. 2014 **[64]	q-RCT	Exercise group (no control group)	Supervised exercise Warm up = 5–10 min walking Main training = 30 min walking or jogging Cool-down = 5–10 min walking and stretching	3 times/week for 10 weeks	Main training jogging duration of 6^th^-10^th^ session: 30 min continuously Main training jogging-walking intervals duration from 1^st^ to 5^th^ session (min): Steady increase in running time compared to walking time through weeks	-	-
	**Varkey et al. 2009 **[66]	q-RCT	Exercise group (no control group	Supervised exercise Warm up = 15 min indoor cycling Main training = 20 min indoor cycling Cool-down = 5 min indoor cycling	3 times/week for 12 weeks	Total duration = 40 min	Warm up = 11–13 Borg Main training = 14–16 Borg Cool-down = 11–13 Borg	-
	**Hagan et al. 2021** [71]	Cohort	Exercise	Unsupervised exercise	0, 1–2, 3–4 or 5 times/week	-	Light: normal walking, walking downstairs, yoga, gardening, etc Moderate: brisk walking, lawn mowing, shoveling, dancing, etc Vigorous: jogging, running, cycling fast, kickboxing, etc	-
**Yoga**	**Boroujeni et al. 2015 **[45]	RCT	Yoga	Supervised exercise -Eye-related exercises -Backward bending exercises -Fist pavanmoktasana -Second pavanmoktasana -Third pavanmoktasana -Pre-pranayama yoga -Standing-sitting and lying out screw position -Neti exercises -Shavasanas or relaxation Training modality: No info	3 times/week for 12 weeks	Total duration = 75 min	-	-
	**John et al. 2007 **[51]	RCT	Yoga	Supervised exercise -Yoga postures = Stretching of neck, shoulder, back muscles followed by relaxation, toning, strengthening, and flexibility -Breathing and Pranayama -Kriya = Jalaneti (nasal water cleansing) followed by Kapalbhanti (forced exhalations)	5 times/week for 12 weeks	Total duration = 60 min	-	-
	**Kisan et al. 2014** [52]	RCT	Yoga + conventional care	Supervised exercise -Relaxation exercises -Breathing exercises -Asanas/posture with awareness -Shavasana Training modality: No info	5 times/week for 6 weeks	Total duration = 60 min	-	-
	**Kumar et al. 2020 **[54]	RCT	Yoga + medical treatment	Supervised exercise -Prayer = 1 min -Breathing exercises = 8 min -Instant relaxation technique = 1 min -Sukshma vyayama = 15 min -Surya namaskar = 3 min -Quick relaxation technique = 3 min -Asanas = 8 min -Savana-yoga = 10 min -Pranayama = 15 min Training modality: No info	3 times/ week for 4 weeks	Total duration = 60 min	-	-
	**Mehta et al. 2021** [58]	RCT	Yoga + Standard Drug Therapy	Supervised exercise Pranayama, Asana and Savasana Training material: No info	Daily for 3 months	Total duration = 40 min	-	-
	**Wells et al. 2021** [68]	RCT	Mindfulness-based stress reduction (standardized training in mindfulness/yoga)	Electronic audio files for home mindfulness/yoga practice	2 h/week for 8 weeks with optional retreat day	Total duration = 30 min	-	-
**Exercise and lifestyle recommendations**	**Bond et al. 2018 **[44]	RCT	Behavioral weight loss	Unsupervised Home-based exercise	5 days/week for 16 weeks	Total duration = Gradually progressed to 50 min of home-based exercise/session	-	-
	**Lemstra et al. 2002 **[55]	RCT	Exercise group	Supervised exercise 18 group sessions of aerobic training, strength training, massage, stress management and dietary lecture (relaxation and behavioural therapy) Training modality: No info	6 weeks with 3 months follow-up	-	-	-
	**Gaul et al. 2011 **[70]	Cohort	Muscular progressive relaxation, aerobic exercise and lifestyle recommendation	Supervised exercise -Headache education = 60 min -Behavioural group session = 90 min -Relaxation training = 60 min -Physical therapy = 60 min -Aerobic ergometer training = 60 min	Sessions applied 5 days/week minimum	Total duration of programme = 5 h and 30 min	-	-
	**Seok et al. 2006 **[72]	Cohort	Lifestyle behaviour modifications, exercise and medication use	Unsupervised exercise Maintaining regular exercise Training modality: No info	Session applied at least 3 times/week minimum	Total duration = 30 min minimum per session	-	-
	**Woldeamanuel et al. 2016** [73]	Cohort	Regular Lifestyle Behaviours cohort	Unsupervised exercise Maintain daily aerobic exercise of any form	Session applied at least 6 months Minimum	Total duration = 20 min minimum	Exercise that raises heart rate	-
**Relaxation techniques**	**Meyer et al. 2016 **[59]	RCT	Progressive Muscle Relaxation	Supervised exercise 16 muscle groups that were slightly tensed and thereafter relaxed Training modality: No info	1 time per week for 6 weeks	-	-	-
	**Minen et al. 2020 **[60]	RCT	Progressive Muscle Relaxation	Unsupervised exercise Smartphone app with Progressive Muscle Relaxation program = 15 min Training modality: No info	2–4 times per week, for 6 weeks with a follow-up of 3 months	Total duration = 15 min	-	-
	**Varkey et al. 2011 **[67]	RCT	Relaxation group	Unsupervised relaxation Relaxation (6 relaxation exercises, each exercise 5–20 min)	Daily	Total duration: 30–120 min	-	-
**High-Intensity aerobic Interval Training**	**Hanssen et al. 2017 **[50]	RCT	HIIT	Supervised exercise Warm-up = 400 m easy running on a treadmill and 2 skipping exercises Main training = High-intensity interval running on a treadmill Cool down = 400 m easy running on a treadmill and stretching exercises	2 times/week for 12 weeks	High-intensity intervals vs active rest period (min) = 4–3 High-intensity intervals were repeated 4 times, with a total duration of 16 min of high-intensity	High-intensity intervals = 90–95% HRmax (± 5 bpm) reached after 1 min from the beginning of the high-intensity interval Active rest intervals = 70% HRmax	-Individual anaerobic lactate-threshold -HRmax -VO_2_ max (supervised)
	**Hanssen et al. 2018 **[49]	RCT	HIIT	Supervised exercise Warm-up = 400 m easy running on a treadmill and 2 skipping exercises Main training = High-intensity interval running on a treadmill Cool down = 400 m easy running on a treadmill and stretching exercises	2 times/week for 12 weeks	High-intensity intervals vs active rest period (min) = 4–3 High-intensity intervals were repeated 4 times, with a total duration of 16 min of high-intensity	High-intensity intervals = 90–95% HRmax (± 5 bpm) reached after 1 min from the beginning of the high-intensity interval Active rest intervals = 70% HRmax	-Individual anaerobic lactate-threshold -HRmax -VO_2_ max (supervised)
	**Matin et al. 2022 **[57]	RCT	High-Intensity Interval Aerobic Exercise, B12 and Magnesium Supplementation Group	Supervised exercise Warm-up = 10 min Main training Cool down = 10 min Training modality: Possibly outdoor and indoor cycling	3 times/week for 8 weeks	Main training from 1^st^ to 8^th^ wk (min): 10, 15, 20, 25, 30, 35, 40, 40 High-intensity interval duration: No info Low-intensity interval duration: No info	High-intensity interval Borg’s rating from 1^st^ to 8^th^ wk: 11, 12, 14, 15, 16, 17, 18, 18 High-intensity interval % VO_2_ max from 1^st^ to 8^th^ wk: 60, 60, 60–65, 65–70, 70–75, 70–75, 75–80, 80 Low-intensity intervals: No info	-VO_2_ max
**Low-intensity aerobic exercise**	**Santiago et al. 2014 **[65]	RCT	Amitriptyline and Aerobic Exercise Group	Unsupervised exercise Warm-up exercises Main training = Fast walking outdoors	3 times/week for 12 weeks	Total duration = 40 min	-	-
	**Köseoglu et al. 2003 **[53]	q-RCT	Exercise group (no control group)	Unsupervised exercise Warm up = 10 min Main training = 20 min aerobic exercise Resting period = 10 min Training material = No info	3 times/week for 6 weeks	Total duration = 40 min	Main training = 60% HRmax	HRmax
**Exercise and relaxation techniques**	**Butt et al. 2022** [46]	RCT	Moderate Intensity Continuous Aerobic Exercise and Progressive Muscle Relaxation Group	Supervised exercise Warm-up = 10 min stationary cycling Aerobic exercise main training = 30 min stationary cycling Cool down = 5 min stationary cycling Progressive muscle relaxation = 15 min	3 times/week for 6 weeks	Total duration = 60 min	-	-
	**Dittrich et al. 2008 **[48]	RCT	Exercise group	No information of supervision -Warm-up = 5 min -Aerobic exercise including training of coordination = 15–25 min -Strength training = 10–20 min -Stretching = 5 min -Progressive muscle relaxation = 15 min	2 times/week for 6 weeks	Total duration = 60 min	-	-
	**Mehta et al. 2021** [58]	RCT	Physiotherapy and Standard Drug Therapy (Relaxation and exercise)	Supervised exercise Progressive muscle relaxation exercise, self-stretching of neck muscles (30 s hold 3 repetitions), isometric exercise of neck muscles (5 s hold; 10 repetitions) and cardiorespiratory endurance training (30 min walking) Training material: No info	Daily for 3 months	Total duration = 40 min	-	-
**Neck strength exercise**	**Bennato et al. 2022** [43]	RCT	Neck strength exercise	First 6 weeks: 2 sets of 10 repetitions with 10 s of endurance for the deep cervical flexor and extensor muscles. Progression in series, repetitions and endurance was based on absence of complaint or pain, and/or execution of movement without compensation in each volunteer Last 2 weeks: add to the previous exercise 3 sets of 15 repetitions for flexor and extensor superficial cervical muscle	2 sessions/day for 8 weeks Supervised by a physiotherapist once a week for 20 min in an individual session. The rest of the program was done at home	-	-	-
**Tai Chi**	**Xie et al. 2022 **[69]	RCT	Exercise group	3 times/week supervised, and 2 times/week unsupervised Warm-up with stretching = 10 min Main training = 45 min. In the first 15 supervised lessons (first 5 weeks), participants learned individual parts of the Modified 33-short form Yang-style Tai Chi Chuan. From the 16th session, participants performed the whole exercise program 3 times per session (15 min each) Cool down with stretching = 5 min Training material = Not needed	5 times/week for 12 weeks Participants in Tai Chi group kept at least 1 session/week in the following 12 weeks after the end of treatment	Total duration = 60 min	-	-
**Resistance exercise**	**Aslani et al. 2021** [42]	RCT	Resistance Training	Supervised exercise Warm up = 5 min jogging, 5 min stretching and 5 min weightlifting Main training = 2–3 sets of 8–15 repetitions of arm pull down, arm pull over, sit up, leg extension, leg curl Cool down = 5 min active cooling and stretching movements Training modality: No info	3 times/week for 8 weeks	Total duration = 30–45 min	Main training performed from 45% RM to 75% RM gradually progressed through the 8 weeks	RM
**Qi-gong**	**Elinoff et al. 2019** [74]	Case series	Qigong Exercise	Supervised exercise -First face-to-face exercise sequence = Ju Fu (Gentle Wind) -Qigong exercise DVD duplicating the content of the first and subsequent lessons for home practice = 10 min minimally -Two subsequent face-to-face sessions to reinforce training and add complexity and length to the Kiko sequence	-Daily home practice -Two subsequent face-to-face sessions every 30 days	Total duration = 10 min at least	-	-

*Abbreviations*: *bpm* Beats per minute, *HIIT* High-Intensity Interval Training, *HR* Heart Rate, *HRmax* Maximal heart rate, *MCT* Moderate Continuous Training, *m/min* Metres/minute, *min* Minute, *q-RCT* Quasi-Randomized Controlled Trial, *RCT* Randomized Controlled Trial, *RM* Repetition Maximum, *VO2max* Maximal oxygen uptake

**Table 11:** Summary of prescription parameters for each exercise modality based on the prescription parameters used in the included studies

Type of intervention	Migraine diagnosis	Trials	Type of exercise	Distribution	Frequency	Duration (per session)	Intensity	Exercise testing	Grade of recommendation
**Moderate intensity continuous aerobic training**	Episodic or chronic migraine	*N* = 12 RCTs (*n* = 6): Hanssen 2018 [49], Varkey 2011 [67], Hanssen 2017 [50], Oliveira 2017 [62], Oliveira 2019 [63], Ahmadi 2015 [41] Q-RCTs (*n* = 5): Darabaneanu 2011 [47], Luedtke 2020 [56], Varkey 2009 [66], Overath 2014 [64], Narin 2003 [61] Cohort (*n* = 1): Hagan 2021 [71]	Supervised modalities: running, jogging, indoor cycling or cross-training Unsupervised modalities: nordic walking, slow running, outdoor cycling, swimming, cycling ergometer, brisk walking, dancing, other activities Supervised exercise (n = 9): Hanssen 2018 [49], Varkey 2011 [67], Hanssen 2017 [50], Oliveira 2017 [62], Oliveira 2019 [63], Darabaneanu 2011 [47], Varkey 2009 [66], Overath 2014 [64], Narin 2003 [61] Supervised and unsupervised exercise (n = 2): Luedtke 2020 [56], Narin 2003 [61] No information concerning supervised/unsupervised (n = 1): Ahmadi 2015 [41]	Warm up from 5 to 15 min with walking, jogging, or easy cycling Main training performed from 20 to 30 min Cool down from 5 to 10 min with easy cycling, jogging, walking or stretching	2–3 times/week for 5–12 weeks	Total duration of 30 to 50 min	Warm up gradually increased from 11 to 13 Borg Main training performed between 13–16 Borg, 70% HRmax (± 5 bpm) or at the intensity corresponding to participant’s ventilatory threshold Cool down between 11–13 Borg	Initial evaluation of individual anaerobic lactate-threshold, HRmax, VO_2_ max or ventilatory threshold (calculated with lactate blood test or respiratory gas exchange analysis) Monitoring during exercise with Borg scale, %HRmax and/or speed (m/min)	B in favour of intervention
**Yoga**	Episodic migraine	*N *= 6 RCTs (6): Kumar 2020 [54], Kisan 2014 [52], Boroujeni 2015 [45], John 2007 [51], Mehta 2021 [58], Wells 2021 [68]	Yoga: Full program under supervision; the first session or first month is supervised and the rest of the program is performed at home with audio-visual guidance if possible, or ensuring compliance with the routine with a telephone call every week or two months and/or with a diary checking compliance or self-reported yoga log maintained by the patient, and/or with the possibility of visiting professionals	First part: Starting prayer, breathing, stretching and relaxation exercise (including Instant Relaxation Technique and Quick Relaxation Technique). Eye-related and backward bending exercise Second part: Asanas, savana, pavanmoktasanas, pranayama or pre-pranayama, neti exercise, standing-sitting and lying out screw position, kriya (Jalaneti followed by Kapalbhanti), sukshma vyayama, surya namaskar Final part: Shavasana or relaxation	3–7 times/week for 6–12 weeks	Total duration of 60–75 min	-	-	B in favour of intervention
**Exercise and lifestyle recommendations**	Episodic and chronic migraine	*N* = 5 RCTs = 2 Bond 2018 [44], Lemstra 2002 [55] Cohort = 3 Seok 2006 [72], Wodeamanuel 2016 [73] Gaul 2011 [70]	Home-based exercise, stretching, light weightlifting training, endurance training (mainly using sport gym equipment), or any modality of daily aerobic exercise that raise the heart rate	-	3–7 times/week for 6 weeks to more than 6 months	Total duration of 20–60 min	Main training performed at a moderate to submaximal intensity	-	B in favour of intervention
**Relaxation techniques**	Episodic and chronic migraine	RCTs (*n* = 3): Varkey 2011 [67], Meyer 2016 [59], Minen 2020 [60]	-	6 relaxation exercises based on breathing and stress-management techniques, from 5 to 20 min of duration each exercise, or Progressive Muscle Relaxation including 16 muscle exercises or Smartphone app with Progressive Muscle Relaxation program	1–6 times/week for 6–12 weeks	Total duration of 15 min to 120 min	-	-	C in favour of intervention
**High-intensity aerobic interval training**	Episodic migraine	*N* = 3 RCTs = 3 Hanssen 2017 [50] Hanssen 2018 [49] Matin 2022 [57]	Running on a treadmill Bicycle Supervised	Warm-up = 400 m easy running on a treadmill and 2 skipping exercises or 10 min cycling Main training = High-intensity interval running on a treadmill or bicycle Cool down = 400 m easy running on a treadmill and stretching exercises or 5 min cycling	2–3 times/week for 8–12 weeks	Main training = 10–40 min High intensity – moderate intensity intervals (min) = 4–3 High-moderate intensity intervals were repeated 4 times	High intensity: Progression from Borg 11 to 18 or from 60% VO_2_ max to 80% in 8 weeks Maximum high-intensity reached 90%-95% HR Max Maximum active rest period intensity reached: 70% HRmax	-Individual anaerobic lactate-threshold -HRmax -VO_2_ max (supervised) -Borg	C in favour of intervention
**Low-intensity aerobic exercise**	Episodic migraine	*N* = 2 RCT (1): Santiago 2014 [65] Q-RCT (1): Köseglu 2003 [53]	Home active exercise or fast walk outdoors, not supervised	Warm-up exercises for 10 min Main training performed for 20–40 min Resting period performed for 10 min	3 times/week for 6–12 weeks	Total duration of 40 min	Main training performed at 60% HRmax	HR	C in favour of intervention
**Exercise and relaxation techniques**	Episodic and chronic migraine	RCT (*n *= 2): Dittrich 2008 [48] Mehta 2021 [58] Q-RCTs (*n* = 1) Butt 2022 [46]	Relaxation exercise and stationary cycling, or gymnastics with music, aerobic and strength training, or stretching, isometric exercise and walking Not reported if supervised or not	Warm up = 5–10 min Main training = 30 min of moderate aerobic exercise or 15–25 min of aerobic training and 10–20 of strength training Or self-stretching of neck muscles (30 s hold 3 repetitions), neck isometric exercise (5 s hold, 10 repetitions) and 30 min walking Progressive muscle relaxation = 15 min Cool down or stretching = 5 min	2–3 times/week for 6–12 weeks	Total duration of 45–60 min	-	-	C in favour of intervention
**Neck strength exercise**	Episodic migraine	RCT (*n* = 1): Benatto 2002 [43]	Strength exercise for superficial and deep flexor and extensor craniocervical musculature with home exercise for craniocervical musculature and stretching	First stage: deep muscle training, 2 sets of 10 repetitions for deep flexor and extensor musculature, for 6 weeks. Individually progressed in number of series, repetitions and endurance Second stage: deep and superficial muscle training for the next 2 weeks, including 3 sets of 15 repetitions for superficial flexor and extensor musculature	1 day per week under supervision and 2 times/day everyday with home exercises for 8 weeks	Total duration of 20 min	-	-	C against the intervention
**Tai Chi**	Episodic migraine	*N* = 1 RCTs (*n* = 1): Xie 2022 [69]	Modified 33-short form of Yang-style Tai Chi Chuan, (including the form “closing”) The protocol included both supervised and unsupervised exercise	Warm-up with stretching for 10 min Main training of 45 min, with the first 5 weeks learning individual exercises of the Tai Chi exercise program The following 6–12 weeks participants perform the whole Tai-Chi exercise program, 3 times per session Cool-down with stretching for 5 min	5 times/week for 12 weeks	Total duration of 60 min	-	-	C in favour of intervention
**Resistance exercise**	Episodic migraine	RCTs (*n* = 1): Aslani [42]	Resistance exercise with dumbbells, arm pull down, arm pull over, sit up, leg curl machine, and leg extension machine Not reported if supervised or not	Warm up for 15 min with jogging, stretching, and weightlifting Main training performed from 30 to 45 min, 2–3 sets of 8–15 repetitions of arm pull down, arm pull over, sit up, leg extension and leg curl Cool down for 5 min with active cooling and stretching movements	3 times/week for 8 weeks	Total duration of 30 to 45 min	Main training gradually performed from 45% RM to 75% RM	RM	C in favour of intervention
**Qi-Gong**	Episodic migraine	Case series (*n* = 1): Elinoff 2019 [74]	Supervised exercise Ju Fu (Gentle Wind) method	First face-to-face history of Qi-Gong explanation and exercise sequence = Ju Fu (Gentle Wind) -Qigong exercise DVD duplicating the content of the first and subsequent lessons for home practice Two subsequent face-to-face sessions to reinforce training and add complexity and length to the Kiko sequence	Daily home practice, for 3 months Two subsequent face-to-face sessions every 30 days	Total duration of 10 min at least	-	-	D in favour of intervention

*Abbreviations*: *bpm* Beats per minute, *HR* Heart rate, *HRmax* Maximal heart rate, *m/min* Meters/minute, *min* Minutes, *q-RCT* Quasi–Randomized Clinical Trial, *RCT* Randomized Controlled Trial, *RM* Repetition Maximum, *VO*_*2*_*max* Maximal oxygen uptake

#### Limitations and future directions

The purpose of this clinical practice guideline is to describe in depth the scientific evidence on exercise prescription for patients with migraine in order to facilitate decision making by physical therapists and other health and exercise professionals. Analysis of the information incorporated herein shows that in the last decade there has been an increase in the number of studies on the effectiveness of exercise in patients with migraine. Most of the research we included presented positive effects; however, there are several limitations in these studies that should be considered when interpreting the results and considering the future direction of studies in this area.

One of the most important limitations of the analyzed and included evidence is related to the comparisons used in the studies: the control groups employed a wide variety of interventions, including waiting lists, placebo, and pharmacological treatments. This limitation is fundamentally derived from ethical requirements, given that pharmacotherapy is established as the first line of treatment, and this situation substantially complicates determining the real magnitude of the effect of the various exercise modalities on migraine.

In relation to the above, it should also be considered that it is not possible to establish a real placebo comparison for treatments in which behavior modification is promoted, as in the case of exercise.

Another limitation is that most of the studies based on exercise and migraine evaluated immediate response, in short and intermediate terms, and only a few studies measured long-term effects. We believe that it is necessary to design studies that assess long-term effects to identify whether the effect is dependent on the duration of the exercise intervention or whether the effect is maintained for a long time after the intervention. At least a one year follow up would be recommended [24]. It would also be necessary to perform analyses that identify the level of adherence to the interventions related to exercise.

When using exercise with migraine patients the question arises as to whether it is appropriate to exercise with very intense pain. There is no evidence about the effects of the exercise while the patient is under severe pain, however we consider we must be cautious when prescribing exercise. If the patient has very intense pain, very frequently and/or the exercise is a clear trigger factor, the most appropriate recommendation would be to prescribe a gradual and individualized exposure to exercise.

There remain some unknowns about the effect of exercise on the patient with migraine that need to be addressed in future research. Studies do not currently compare which exercise modality is most effective in reducing the frequency, duration, and intensity of migraine pain, and it would be interesting to identify whether integrating various exercise modalities (e.g., aerobic exercise and strength training) is more effective than using each modality individually.

The evidence currently available does not clarify whether the improvement of physical variables through exercise has an impact on the frequency, duration, and intensity of migraine. It would be necessary to perform studies that introduce as covariates the level of physical activity, strength, or the improvement of range of motion or cardiovascular capacity and determine whether these variables are associated with the improvement of clinical variables.

Finally, we consider it opportune that subsequent studies take into account the psychological status of the patients to better select the most appropriate exercise modality, due to the growing number of studies that point out the impact that kinesiophobia has on patients with migraine [94, 104, 105]. Considering this factor, prescribing an exercise modality, such as gradual exposure to exercise, may be the most appropriate selection for this patient profile.

## Conclusions

This clinical practice guideline has followed a rigorous process of quality assessment of the scientific evidence related to the effectiveness of exercise on migraine. Our analysis indicates that aerobic exercise, moderate intensity aerobic exercise, yoga, and lifestyle recommendations that include exercise present a grade B of recommendation for reducing the frequency, duration, and intensity of pain and improving the quality of life in patients with migraine.

The exercise modalities that are effective and have a grade C of recommendation are relaxation techniques, interval training at high intensity, continuous low-intensity aerobic exercise, Tai Chi, and resistance training (strength training). Finally, grade D of recommendation was given to Qi-Gong.

## Supplementary Information

Additional file 1. 
