# Supplementary material for: Prescription of therapeutic exercise in migraine, an evidence-based clinical practice guideline
Source: J Headache Pain. 2023 Jun 7;24(1):68. doi: 10.1186/s10194-023-01571-8 (PMC10245624; doi:10.1186/s10194-023-01571-8)
Supplement: Supplementary file 1 — Additional file 1. [file 10194_2023_1571_MOESM1_ESM.docx]

**Search strategy for each database.**

**PubMed**

("Migraine Disorders"[MeSH Terms] OR "migraine"[All Fields]) AND ("Exercise"[MeSH Terms] OR "Exercise"[All Fields] OR "Exercise Therapy"[MeSH Terms] OR "exercise therap*"[All Fields])

("Migraine Disorders"[MeSH Terms] OR "migraine"[All Fields]) AND "Yoga"[All Fields]

("Migraine Disorders"[MeSH Terms] OR "migraine"[All Fields]) AND "Tai Chi"[All Fields]

("Migraine Disorders"[MeSH Terms] OR "migraine"[All Fields]) AND ("Qigong"[All Fields] OR "KIKO"[All Fields])

("Migraine Disorders"[MeSH Terms] OR "migraine"[All Fields]) AND ("resistance exercise"[All Fields] OR "resistance training"[All Fields] OR "strength exercise"[All Fields] OR "strength training"[All Fields])

("Migraine Disorders"[MeSH Terms] OR "migraine"[All Fields]) AND ("relaxation"[All Fields] OR "breathing"[All Fields])

("Migraine Disorders"[MeSH Terms] OR "migraine"[All Fields]) AND "lifestyle"[All Fields] AND "exercise"[All Fields]

*Filters: Systematic Review; Meta-Analysis; Observational Study; Clinical Trial; Randomized Controlled Trial; Case Reports; Review; Adult: 19+ years; Humans.

**PEDro**

Abstract & Title: migraine exercise

Abstract & Title: migraine yoga

Abstract & Title: migraine / Therapy: fitness training

Abstract & Title: migraine / Therapy: strength training

Abstract & Title: migraine exercise / Therapy: behavior modification

**Cochrane**

(migraine) AND (exercise)

(migraine) AND (yoga)

(migraine) AND (tai chi)

(migraine) AND ((qigong) OR (kiko))

(migraine) AND ((resistance exercise) OR (strength exercise))

(migraine) AND ((relaxation) OR (breathing))

(migraine) AND (lifestyle) AND (exercise)

**EBSCO**

TI migraine AND exercise

TI migraine AND yoga

TI migraine AND Tai Chi

TI migraine AND Qigong OR TI KIKO

TI migraine AND TI resistance exercise OR TI strength exercise

TI migraine AND TI relaxation techniques OR TI breathing exercises

TI migraine AND TI lifestyle AND exercise

*Filters: all adult: 19+ years

**Google Scholar**

allintitle: migraine aerobic OR exercise

allintitle: migraine yoga

allintitle: migraine Tai Chi

allintitle: migraine Qigong OR KIKO

allintitle: migraine resistance OR strength

allintitle: migraine relaxation OR breathing

allintitle: migraine lifestyle

**Web of Science**

(TS=(migraine)) AND TS=(exercise)

(TS=(migraine)) AND TS=(aerobic exercise)

(TS=(migraine)) AND TS=(Yoga)

(TS=(migraine)) AND TS=(Tai Chi)

((TS=(Qigong)) OR TS=(KIKO)) AND TS=(migraine)

((((TS=(resistance exercise)) OR TS=(resistance training)) OR TS=(strength exercise)) OR TS=(strength training)) AND TS=(migraine)

((TS=(relaxation)) OR TS=(breathing)) AND TS=(migraine)

((TS=(lifestyle)) AND TS=(exercise)) AND TS=(migraine)

*Filters: Articles; Humans; Migraine Disorders
